# Diffusion-guided 4D microprinting of soft microactuators

**DOI:** 10.1038/s41467-026-73035-x

**Published:** 2026-05-14

**Authors:** Wei-Ting Hsu, Po-An Tsou, Hsin-Jung Chou, Tsung-Kai Lin, Yu-Chieh Cheng

**Affiliations:** Department of Electro-Optical Engineering, National Taipei University of Technology (Taipei Tech), Taipei, Taiwan (R.O.C.)

**Keywords:** Polymers, Actuators, Polymer characterization, Liquid crystals

## Abstract

Four-dimensional (4D) printing has emerged as a powerful strategy for creating reconfigurable soft actuators with applications in biomedical engineering and microrobotics. However, most existing methods, such as inkjet printing or photoalignment, are restricted to film-like or net-like architectures that only enable out-of-plane deformation and cannot be scaled to the microscale. Although two-photon polymerization provides submicron resolution and 3D microfabrication, its voxel-by-voxel laser scanning induces strong liquid crystal diffusion around each printed voxel, which cannot be effectively suppressed by external fields or laser-induced anchoring. Here, we harness this diffusion as a design principle for 4D microprinting of soft liquid crystal network microactuators, enabling hierarchical and volumetric alignment within complex 3D architectures. Diffusion-guided orientation emerges perpendicular to the printed surface profile, allowing programmed molecular alignment through controlled variation of the scanning direction. These microactuators incorporate volumetrically programmable 3D architectures and can be integrated into multi-jointed microarms and microgrippers, executing complex and coordinated actuations for advanced microrobotics.

## Abstract (web summary)

Four-dimensional printing methods are promising for soft actuators, but existing methods limit programmable molecular alignment to two dimensions. Here the authors report a diffusion-guided two-photon polymerization strategy achieving true volumetric 3D microprinting of liquid crystal network microactuators, enabling complex actuation.

## Introduction

Smart materials have attracted increasing interest in biomedical applications, where microscale miniaturization enables compact, stimuli-responsive systems capable of programmable deformation^1^. While semiconductor lithography can produce rigid microstructures^2,3^, soft polymeric materials such as liquid crystal networks (LCNs)^4–6^, including liquid crystal elastomers (LCEs), offer excellent biocompatibility, making them wellsuited for applications such as cell engineering and tissue scaffolding^7,8^. Beyond their softness and biocompatibility, LCNs doped with nanoparticles^9–12^, azobenzene^13,14^, or photothermal dyes^15^ exhibit light- or heat-induced reversible deformations, giving rise to diverse motions^16–24^. By predefining the liquid crystal (LC) director field, diverse motion modes can be programmed into soft robotic actuators^25,26^. However, most systems remain film-like and lack three-dimensional (3D) structural complexity. Efforts to overcome this limitation include patterned topologies^27–29^, origami-inspired designs^23,29^, and 4D printing^30–33^, which generate out-of-plane deformations starting from planar, film-like, or net-like precursor forms. However, realizing 4D microprinting of microscale 3D bulk architectures remains challenging.

Two-photon polymerization (TPP), also referred to as direct laser writing (DLW), offers a powerful route for fabricating 3D microscale architectures^34,35^ and has recently been extended to the development of LCN-based microactuators and microrobots^36–39^. However, most LC alignment strategies integrated with TPP for programmable 4D shaping still depend on pre-patterned alignment layers on the substrate^40,41^ or along microscaffolds^42^, which inherently limit the degree of freeform alignment control. Consequently, these approaches are not truly capable of full 4D shape programming. Although magnetic^43^ and electric fields^44,45^ can induce LC alignment without prefabricated layers, their applicability in TPP-fabricated 3D microstructures is limited because they typically enable only binary switching between horizontal and vertical states and require a non-negligible response time for mesogen reorientation. Furthermore, the presence of polymerized structures can distort local electric fields, making precise voxel-level alignment during TPP fabrication far less feasible.

Recent breakthroughs have demonstrated that laser scanning trajectories can dictate molecular alignment in LCN thin films^46,47^, and this capability was subsequently extended to filamentary microstructures written in three dimensions via TPP^48^. However, such control remains restricted to isolated segments or planar geometries. While Directional Tuning Mode (DiTuM)^49^ attempted spatially continuous volumetric control, its dependence on a competing balance between laser-induced alignment axes and initial anchoring results in continuous gradients rather than abrupt orthogonal orientations. This dependency on scanning trajectories precludes precise volumetric tensor control, leaving volumetric 4D microprinting with programmable, arbitrary 3D director fields as an unresolved challenge.

Beyond LCN-based microstructures, all soft microrobots suffer from a fundamental trade-off between geometric complexity and actuation density. Specifically, generating fundamental motions such as bending or twisting necessitates a precisely programmed orthogonal orientation field within the material’s cross-section. This requirement is exceptionally difficult to satisfy at the microscale. While pioneering work in micro-origami and thin-film actuators can achieve high structural precision through sophisticated semiconductor fabrication^2,3^, these platforms are often constrained by their surface-dominated architectures. Consequently, achieving complex, multi-axis activation typically requires dense arrays of discrete hinges and control circuitry, which increase the mechanical overhead and limits deployment within confined geometries. To achieve high functional density within a soft, monolithic body, 3D volumetric programming, which embeds actuation logic directly into the molecular orientation field, represents a powerful paradigm shift. Nonetheless, realizing such a capability remains a formidable challenge, as current fabrication platforms struggle to encode the required orthogonal gradients in situ without laborious post-fabrication manipulations.

Here, we report a diffusion-guided 3D volumetric printing strategy that overcomes microscale limitations in size and architectural complexity by directly embedding high-density actuation logic into a dynamically evolving molecular field. Specifically, we harness LC monomer diffusion through layer-by-layer TPP architectures to encode spatially programmed alignment, thereby enabling volumetric 4D microprinting of soft microactuators. We show that molecular orientation continuously realigns perpendicular to the advancing printed surface profile. At the high scanning speeds used in DLW, laser-induced anchoring is insufficient to override the diffusion-driven orientation process. This mechanism enables progressive, volumetric control of the LC director field, mimicking the seamless integration of form and function in biological morphogenesis and allowing monolithic microactuators with embedded actuation logic to perform coordinated, multi-degree-of-freedom motions.

## Results and discussion

### Diffusion-driven sidewall alignment via DLW for seamless in-plane LCN layers

**Fig. 1:**
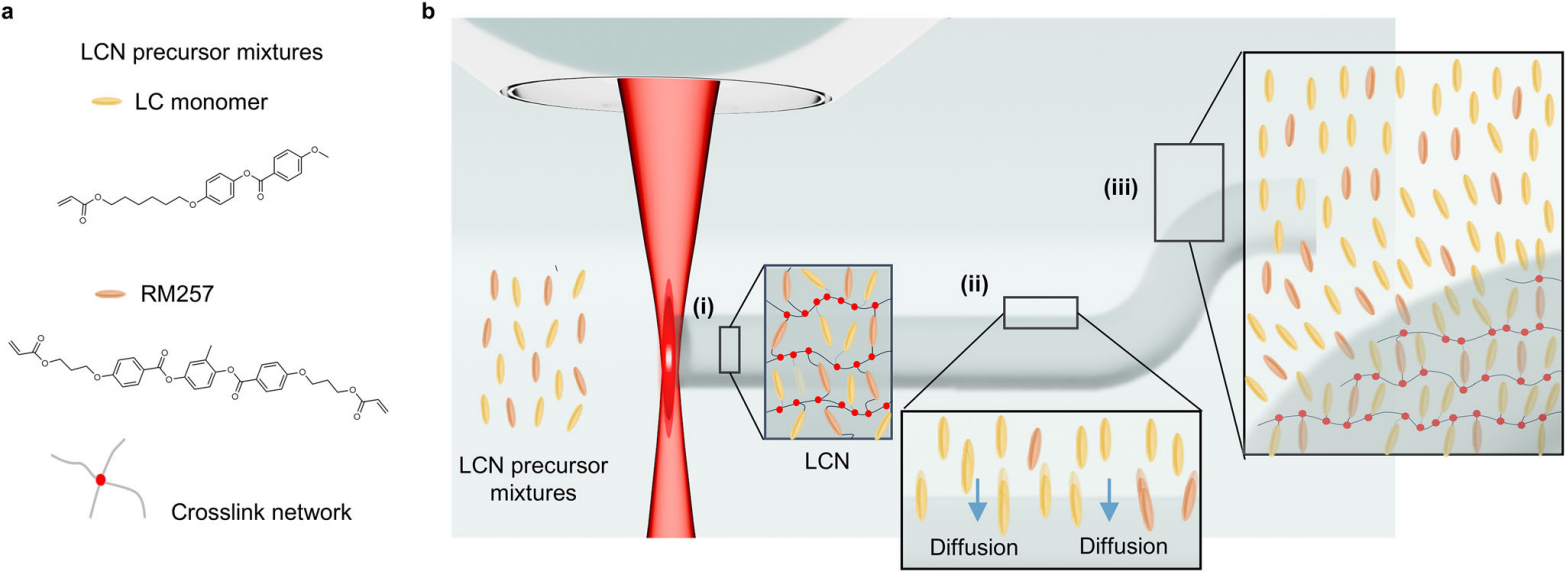
Schematic illustration of diffusion-driven reorientation via TPP. **a** Chemical structures of the precursor mixture, consisting of LC monomers (yellow ellipses) and RM257 crosslinkers (orange ellipses), which form a crosslinked network (red junctions) upon photopolymerization. **b** LC monomers initially align vertically due to the untreated glass substrate. The red voxel represents the focal volume of the femtosecond laser. **b**(i) Polymerization fixes the alignment within the voxel. **b**(ii) LC monomers (ellipses) diffuse into the LC network. **b**(iii) Near the polymer facet, LC mesogens reorient perpendicular to the surface, producing spatially varying alignment that follows the voxel-line profile, before returning to the alignment dictated by the substrate anchoring.

The concept of diffusion-guided alignment during TPP is illustrated in Fig. 1. A femtosecond laser rapidly scans through the LCN precursor mixture (Fig. 1a), polymerizing only along the voxel-scale writing path. The unpolymerized LC monomers at the interface remain mobile and, driven by diffusion, reorient into a perpendicular alignment, as shown in Fig. 1b. At a high scanning speed (2200 μm s⁻¹), mesogen orientation within the voxel region is rapidly immobilized due to the fast polymerization kinetics (Fig. 1b, (i)). This process locally consumes reactive monomers, thereby establishing a sharp spatial gradient in chemical potential at the polymer/precursor interface. The surrounding mobile LC monomers redistribute toward the interface and partially into the newly formed network region, driven by local chemical-potential gradients arising from local differences in monomer density (Fig. 1b, (ii)). During this transport process, LC mesogens near the interface realign to minimize interfacial energy, following the surface contour to form a spatially graded orientation (Fig. 1b, (iii)). While this diffusion-induced realignment is localized near the polymer interface, LC mesogens further from the structure gradually relax into the equilibrium state dictated by the substrate’s global anchoring.

**Fig. 2:**
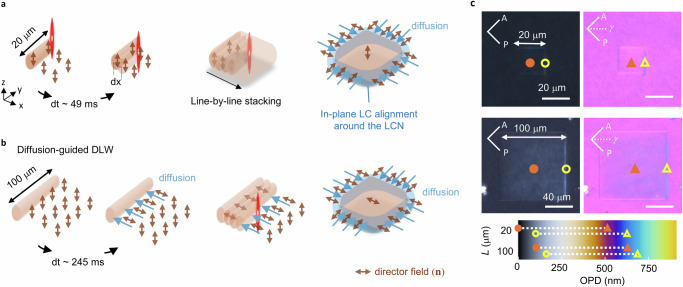
Diffusion-guided alignment of the single-layer LCNs. **a** For an LCN layer composed of short line segments (20 μm), the writing duration per line is approximately *dt* = 49 ms. The orange regions represent the TPP-polymerized LCN, while the brown arrows indicate the director field. Each subsequent line partially overlaps the previous one with a lateral step size of *dx* = 0.2 μm. The surrounding unpolymerized precursor maintains vertical alignment due to substrate anchoring, whereas the edges of the whole LCN layer exhibit in-plane mesogen alignment, oriented perpendicular to the sidewalls**. b** When printing longer line segments, the extended inter-line delay facilitates sidewall diffusion, driving mesogen reorientation from vertical to horizontal and resulting in in-plane alignment. **c** POM images of 20 μm-long and 100 μm-long LCN layers. Under POM with a full-wave (*λ*) plate (right), the right and left edge regions appear blue, while the upper and lower edge regions appear yellow. The dashed line (*γ*) indicates the slow axis direction of the *λ*-plate. At the right edge of the LCN layer, the POM color (yellow dots) shifts from gray to blue after inserting a *λ* = 550 nm retarder with its slow axis (*γ*) horizontal, confirming that the LC director at the edge is parallel to *γ* and thus perpendicular to the edge. The lateral dimension *L* of the structure is 20 μm or 100 μm. A and P denote the analyzer and polarizer, respectively.

To construct either a 2D planar sheet or a 3D volumetric LCN microstructure, voxel line segments are sequentially polymerized with partial overlap. For example, scanning along the y-axis with a lateral step (hatch) of 0.2 μm along the x-axis forms a continuous, structurally coherent LCN layer (Fig. 2). This partial overlap ensures geometric continuity, while the time interval between completing one line and writing the next (the inter-line interval) determines whether LC monomers have sufficient time to diffuse and reorient. For short scan lengths (e.g., a 20 μm line), this inter-line interval is brief, so diffusion during writing is limited. Consequently, the fast polymerization kinetics dominate, trapping the mesogens in their initial vertical alignment dictated by the substrate anchoring, consistent with conventional DLW (Fig. 2a). In contrast, increasing the printed line length effectively prolongs the inter-line delay, enabling LC mesogen diffusion toward the voxel sidewalls. Such a sidewall (single-segment) interfacial diffusion occurs along the surface of a single written line segment (i.e., within one voxel-scanning pass). These diffused mesogens subsequently reorient and are polymerized during DLW, yielding single-layer LCN sheets with in-plane alignment (Fig. 2b). The final director field is determined by the competition between the time required for diffusion-guided reorientation and the arrival time of the subsequent laser pass. Within this same timescale framework, the scan-direction shear from laser-induced anchoring is unlikely to compete with diffusion-guided alignment, because diffusion/reorientation proceeds over micron-scale distances on milliseconds to seconds timescales, whereas local laser exposure and polymerization occur on sub-millisecond timescales (see Supplementary Note 8).

To validate diffusion-guided alignment toward the sidewalls of each written voxel line, we performed birefringence color analysis to determine whether LC mesogens reoriented during varied inter-line delay in DLW and to resolve their orientations at the voxel edges after polymerization. To accurately visualize birefringence colors for LC alignment analysis, the entire LC cell was uniformly UV-cured after DLW (see Supplementary Fig. 1 and Methods). Polarized optical microscopy (POM) images reveal that insufficient diffusion time during voxel-line overlap in the DLW process results in vertical alignment, as evidenced by the dark appearance of the 20 μm-long LCN layer under POM. In contrast, a longer inter-line delay (e.g., the 100 μm-long layer corresponding to ~245 ms) results in a distinct birefringence contrast, indicating in-plane alignment.

To further identify the orientation of this in-plane alignment, a full-wave (*λ* = 550 nm) retarder was inserted with its slow axis aligned horizontally (*γ*, dashed line in Fig. 2c). Depending on whether the mesogen slow axis is parallel or perpendicular to *γ*, the observed POM colors shift due to constructive or destructive retardation, enabling precise orientation determination^50^. For example, in the 100 μm-long LCN layer, the color shifts from light gray to blue under POM with a full-wave retarder, indicating constructive retardation of the optical path difference (OPD, +550 nm) and confirming in-plane alignment parallel to the slow axis of the retarder. A similar blue shift is observed at the right edge, demonstrating that LC mesogens there align perpendicular to the sidewall of the LCN layer. This also indicates that when subsequent voxel line segments are overlapped at the right edge of the LCN layer during DLW, an in-plane LCN domain aligned along the x-axis can be obtained. More quantitative OPD analyses for varying lateral sizes are summarized in Supplementary Fig. 2. Although our configuration illustrates alignment along the x-axis, the approach is readily adaptable to other directions. For example, in-plane alignment along the y-direction can be achieved simply by rotating the overlapping voxel paths, i.e., performing voxel scanning along the x-direction promotes mesogen diffusion toward the sidewalls of the polymerized lines, resulting in LC alignment along the y-direction. This interpretation is further supported by the yellow color observed under POM +* λ* at the upper and lower edges, where LC alignment is perpendicular to the *λ*-plate axis, resulting in reduced retardation and a yellow shift relative to the light gray under POM.

### Dual alignment of bulk LCN via layer stacking

**Fig. 3:**
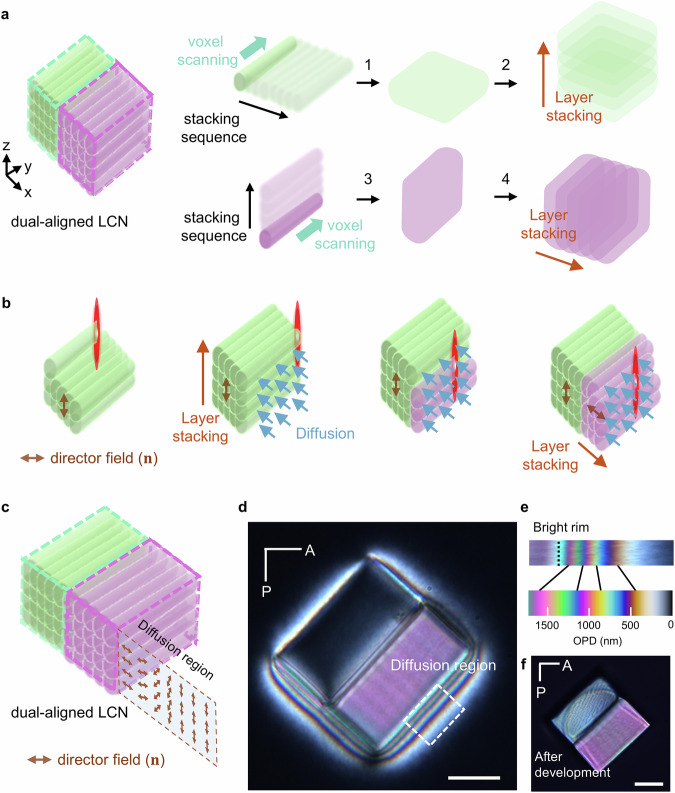
Dual-aligned LCN micropillars via diffusion-guided technique based on layer-stacking. **a** Schematic of the layer-by-layer stacking sequence used to induce horizontal (pink) alignment by combining orthogonal stacking directions. **b** Schematic of diffusion-induced alignment during voxel scanning as the stacking direction changes. **c** Schematic of the resulting dual-domain architecture with orthogonal alignments and the diffusion-influenced interfacial region. **d** POM image of the dual-aligned micropillar before development. **e** Magnified view of the dashed region in d, showing a gradual birefringence color transition consistent with the reference Michel-Lévy sequence. **f** The developed LCN micropillars under POM. Scale bars, 10 μm. A and P denote the analyzer and polarizer, respectively.

Once fabrication proceeds by upward layer stacking to form 3D microstructures, the dominant templating interface shifts from the sidewall of an isolated voxel-scanned line segment to the outer polymer/precursor interface of the already-built multilayer architecture. Accordingly, mobile LC monomers near this evolving interface are reoriented by interfacial templating during diffusion-guided equilibration. Consequently, in volumetric structures, diffusion-guided alignment follows the layer-stacking axis rather than the lateral sidewalls of the voxel-writing line segments. Figure 3a illustrates the orthogonal layer-stacking sequence used to fabricate a dual-aligned LCN structure. The green domain is first written by stacking layers vertically without an inter-layer delay, as its intended orientation matches the substrate-imposed alignment. The pink domain is subsequently written on the sidewall of the green domain, with an inter-layer delay introduced to allow diffusion-guided reorientation into the horizontal alignment (Fig. 3b). This transition is not a discrete mechanistic switch: in both regimes, alignment is templated at the polymer/precursor interface, with the effective templating surface progressively evolving from the boundary of an isolated line segment to the outer surface of a multilayer 3D architecture. Accordingly, the layer-by-layer stacking time, set by the overall design and structure size, should exceed the characteristic diffusion time, so that diffusion-driven reorientation can equilibrate before the next layer is written, ensuring consistent diffusion-guided alignment upon subsequent stacking.

The resulting dual-aligned pillar exhibited two well-defined birefringence domains under POM: a vertically aligned region appearing green and a horizontally aligned region appearing pink (Fig. 3c). Because the pillar thickness is fixed at 12 μm, the observed birefringence colors directly correspond to the LC director orientation. For example, the pink region corresponds to an OPD of ~1610 nm (Fig. 3d), yielding an effective birefringence of Δn ≈ 0.1375 for a 12 μm-thick LCN layer. Near the interface with the bulk LCN, the diffusion-affected edge region shows a gradual birefringence color shift that follows the Michel-Lévy interference-color sequence, consistent with the expected OPD progression (Fig. 3d). It indicates that the diffusion-driven reorientation originates at the sidewalls of the polymerized structure, where the concentration gradient and interfacial anchoring are strongest, and progressively decays with increasing distance from the surface. The mesogens farther from the sidewall relax back toward the substrate-imposed (vertical) anchoring. We also note that the post-TPP UV flood cure used to stabilize the sample may induce boundary OPD variations (e.g., the OPD dip at the edge of the LCN micropillar under POM in Fig. 3e). Importantly, this rim is absent in the developed samples (Fig. 3f) and therefore the alignment disturbance at the edge is negligible. In the developed sample, the vertical region of the dual-aligned pillar appears gray under POM because it is written without the inter-layer delay, yielding a lower effective crosslinking density that is more susceptible to development-induced wash-out and slight distortion, which increases the local OPD, causing the region to appear gray or bright rather than dark. Applying the same inter-layer delay to the vertical region is recommended to preserve geometric accuracy, at the cost of slightly longer writing time.

For switching the alignment away from the substrate-imposed state in a 3D bulk build, an inter-layer delay of >3 s is required (Supplementary Fig. 3). This longer timescale, compared with the single-layer in-plane case in Fig. 2, arises because upward voxel stacking introduces overlap in both lateral and vertical directions, producing a denser, more highly crosslinked network that suppresses mesogen transport and thus slows diffusion-guided reorientation. Nevertheless, this inter-layer delay of more than 3 s does not substantially increase the overall fabrication time, as it corresponds to the laser’s return path after completing an entire layer scan rather than an additional pause before each layer is written. The thermally responsive behavior of dual-aligned LCN-based microstructures is provided in Supplementary Fig. 4 and Supplementary Movie 1, demonstrating that the bending micropillars can be fabricated with programmable multi-directional actuation. Beyond enabling mechanically programmable actuators, the same dual-alignment, diffusion-guided stacking concept can also provide tunable spatial patterning of in-plane director fields in continuous LCN films, with potential relevance to optical functionalities such as local retardation-axis control. As a proof of concept, we patterned a honeycomb lattice in a 2 μm-thick cell to demonstrate localized control of in-plane alignment in a micrometer-thick LCN film (Supplementary Fig. 5).

### Light-driven 4D microprinting of suspended LCN-based microstructures

**Fig. 4:**
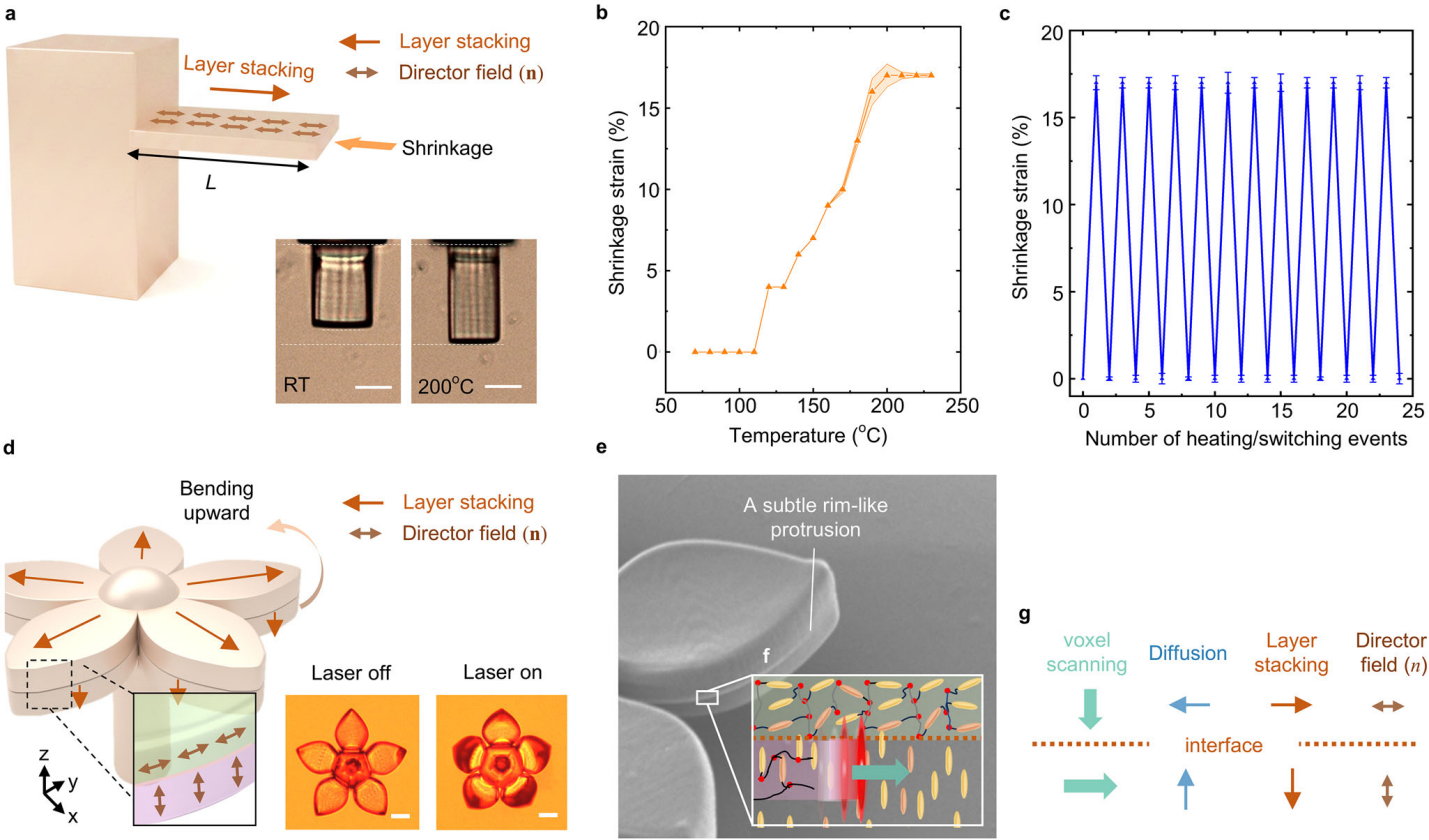
Thermal actuation of horizontally aligned LCN microstrips and 4D microprinting flower-like microstructures. **a** Schematic and optical microscopy (OM) images of a freestanding LCN-based microstrip with horizontal alignment along its longitudinal axis (brown arrow). Upon heating to 230 °C, the microstrip undergoes progressive contraction, reaching a maximum contraction strain of ~17%. **b** Thermal actuation behavior of the freestanding LCN microstrip. **c** Mechanical stability and repeatability of the LCN microactuator (22 mW) under thermal cycling from RT to 200 °C and back to RT. **d** 3D-rendered image of an LCN-based flower featuring a central stem and five suspended radial petals capable of upward bending and OM images of the flower before and after 515 nm laser irradiation, demonstrating light-driven bending. **e** Enlarged SEM view revealing a subtle rim-like protrusion at the interface between the two orthogonally aligned layers, caused by volumetric shrinkage mismatch during development. **f** Schematic of DLW at the interface: the green region represents the first-written polymerized domain, while LC mesogens from the surrounding unpolymerized region diffuse into the green domain and are subsequently polymerized by line-by-line laser scanning (pink color). **g** Schematic summarizing the directions of voxel scanning, mesogen diffusion, layer stacking, and director field orientation for fabricating orthogonal dual-aligned layers. Scale bars, 20 μm. Error bars represent the mean ± standard deviation (*n* = 3), obtained from repeated measurements of the same sample. The lateral dimension *L* of the structure is 55 μm.

To assess mechanical performance and the capability of TPP to fabricate alignment-programmed 3D structures in free space, we fabricated a suspended LCN microstrip that exhibits pronounced stimulus-induced contraction. The structure was produced by DLW at a TPP writing power of 22 mW, with layer stacking along the longitudinal axis to template horizontal mesogen alignment (as illustrated in Fig. 4a). After development, a freestanding LCN microstrip with dimensions of 55 μm in length, 30 μm in width, and 8 μm in thickness was obtained. Upon heating to 230 °C, the microstrip exhibited rapid and reversible contraction along the mesogen alignment direction, with the contraction strain remaining at ~17% above 200 °C (Fig. 4b). To enable photothermal actuation (see Methods), we introduced an azo-dye-based additive after development. The contraction of the suspended microstrip (Supplementary Movie 2) demonstrated a rapid response time under photothermal actuation, reaching an apparent steady-state strain within 50 ms as shown in Supplementary Fig. 6a-c. The shrinkage strain rises to a quasi-steady plateau within tens of milliseconds (typically 40–60 ms at higher excitation powers). After laser turn-off, the strain relaxes back toward baseline within <100 ms. The results in Fig. 4c and Supplementary Fig. 7b show that the shrinkage strain remains remarkably consistent at approximately 17% over 24 consecutive heating/switching events (12 heating/cooling cycles) for different TPP writing powers. This high level of stability demonstrates that the programmed alignment and resulting mechanical work are fully reversible and durable, making these actuators suitable for long-term functional applications.

The TPP writing dose serves as a single, practical control parameter that jointly governs activation characteristics, mechanical integrity, and diffusion-guided alignment. As shown in Supplementary Fig. 7, increasing the TPP writing power (16–28 mW) increases the effective crosslinking density of the printed network, raising ${T}_{g}$ and shifting the onset temperature ${T}_{{on}}$ of the LCN microstrips to higher values. Within this printable/processable dose window, the achieved ~17% actuation strain falls within the reported strain envelope of LCN microactuators, indicating performance comparable to state-of-the-art benchmarks^51^. Further increasing contraction may be possible using longer-chain monomers, but would require careful dose calibration to balance crosslink density and ${T}_{g}$ without compromising print stability. Conversely, reducing dose/voxel size can preserve diffusion-guided reorientation in principle, yet insufficient overlap and stiffness can destabilize 3D stacking, and at extreme reductions polymer conversion may become too weak to sustain the concentration gradients needed for diffusion-driven influx and interfacial reorientation.

We further demonstrate that diffusion-guided alignment enables programmable 3D shape morphing beyond simple contraction, achieving in situ 4D microprinting (Fig. 4d). Each petal integrates dual-aligned domains that together program complex out-of-plane deformation and multidirectional bending. The central stem of the microprinted flower was fabricated by vertically stacking layers along the z-axis without an added inter-layer delay, providing robust anchoring to the substrate for mechanical stability. The petals were then constructed in two sequential steps: lateral stacking in the xy-plane to define radially in-plane alignment, followed by vertical stacking (orange arrows) beneath the petal to obtain the vertical alignment. To further enhance mechanical stability and ensure reliable stitching across orthogonal alignment domains, we intentionally add 3–5 extra overlapping interlayers after switching the stacking direction during fabrication of the suspended petals. Under 515 nm laser irradiation at 11.8 mW, pronounced light-driven 4D shape transformations were observed, as shown in Fig. 4d and Supplementary Movie 3. The observed upward bending independently validates this diffusion-guided control, confirming that the upper layers are horizontally aligned while the lower layers maintain vertical alignment. Supplementary Movie 3 also shows that contraction occurs when the lower layer is not fabricated and the petals contain only in-plane alignment.

Notably, a subtle rim-like protrusion was observed at the mid-plane interface after development (Fig. 4e). This protrusion arises from residual stress release caused by a pronounced volumetric shrinkage mismatch at the interface between two distinct polymer network alignments. Figure 4f provides an enlarged schematic of the diffusion behavior at the interface of a dual-aligned petal. LC monomers diffuse into the upper region of the already built petal, and subsequent voxel-level DLW incorporates these diffused components, thereby establishing a vertically aligned domain in the underlying (bottom) layers. Figure 4g summarizes the directions of voxel scanning, diffusion, and layer stacking during DLW fabrication of suspended LCN petals. Here, the voxel scanning refers to the prescribed scanning path of the DLW focal volume (i.e., the laser voxel) during fabrication.

**Fig. 5:**
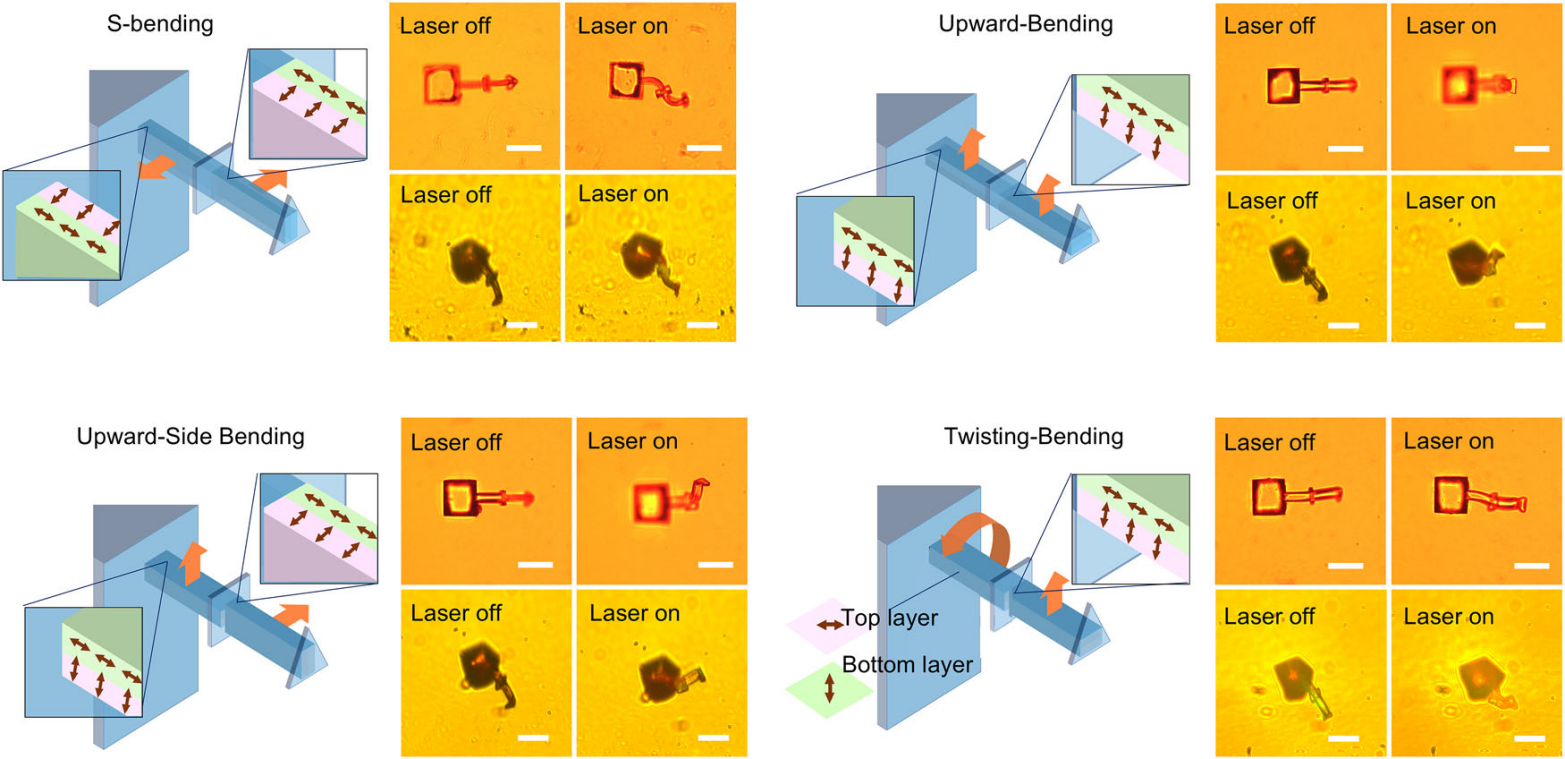
Photothermal actuation and programmable multiaxis deformation modes of multisegment LCN-based microrobotic arms. Schematic illustrations and OM images of microrobotic arms exhibiting programmable deformation modes: S-shaped bending, upward-bending, combined upward-side bending and twisting-bending. Each microrobotic arm comprises multi-segment architectures with spatially encoded LC director fields (brown arrows). In the schematic, the green segments represent regions written first during DLW, while the pink segments indicate areas polymerized last. Scale bars represent 50 μm.

To further demonstrate the versatility of programmable shape morphing, we fabricated a series of freestanding microrobotic arms that exhibit multidirectional actuation, including bending and twisting (Fig. 5 and Supplementary Movie 4). Each microrobotic arm comprises two dual-aligned micropillars whose local director orientations are defined by the stacking direction during TPP. In brief, the initial layers are stacked parallel to the sidewall of a supporting pillar, establishing an in-plane director perpendicular to that sidewall (green inset), and a subsequent orthogonally programmed layer is introduced beneath/adjacent to create an in-plane orthogonal orientation (pink inset), enabling multi-axis deformation under light stimulation. The square-cell junction acts as a compliant transition between neighboring segments, reducing interfacial strain mismatch and improving load transfer and mechanical robustness. More complex behaviors, such as twisting, are achieved by introducing a slanted stacking interface that programs crossed in-plane alignments via ±45° stacking of the upper and lower layers (Supplementary Fig. 8 and Supplementary Movie 5).

**Fig. 6:**
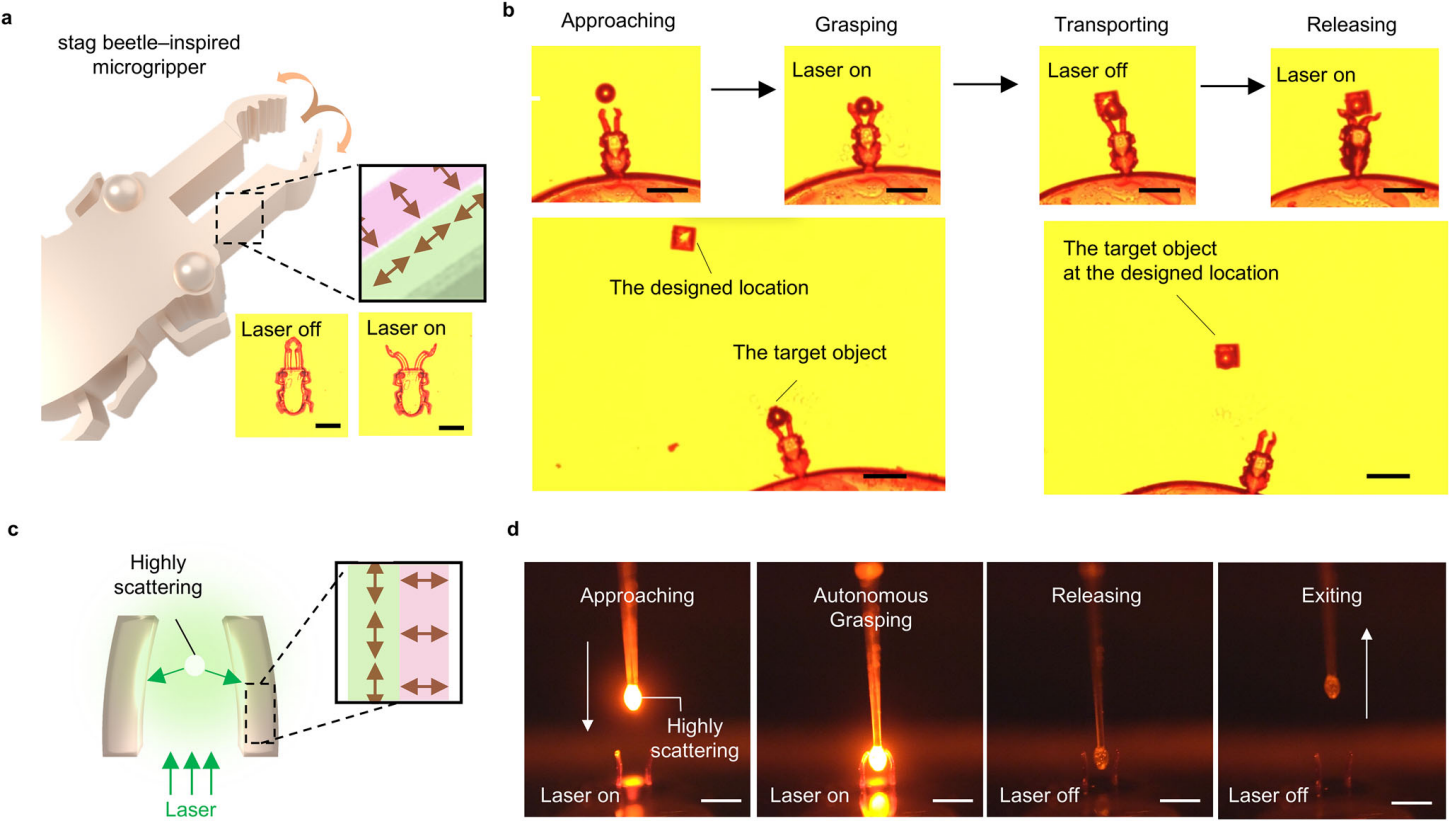
Light-driven biomimetic LCN microgrippers for micromanipulation. **a** Illustration of the stag beetle-inspired microgripper, highlighting the spatial programming of molecular alignment within each mandible. Inset illustrates the stitched alignment domains that program bending. Two adjacent segments (pink and green) are written/stacked with different effective stacking directions, yielding distinct in-plane director fields (brown arrows). Optical images showing the photothermal, reversible deformation of a stag beetle-inspired LCN microgripper, manifested as light-induced jaw opening. Scale bars: 50 μm. **b** Sequential optical images demonstrating microrobotic manipulation under light stimulation. Scale bars: 100 μm. **c** Schematic illustrations of a self-activated, target-triggered LCN microgripper integrated on a cell-culture cavity slide. A bottom-up excitation green laser beam is aligned through the gap between the two LCN pillars, and a highly scattering target locally enhances optical scattering near the gap to trigger closure. Inset illustrates the locally programmed alignment segments that generate the bending/closing response. **d** A time-lapse sequence demonstrating autonomous grasping. Scale bars: 500 μm.

The layer-by-layer stacking strategy enables fully 3D architectures to be fabricated on non-planar substrates, allowing programmable microstructures to be integrated onto tilted or curved geometries as functional interfaces reminiscent of biological cilia or sensory tentacles; for example, longitudinal stacking yields a snail-inspired actuator that mimics head retraction (Supplementary Fig. 9a and Supplementary Movie 6). A wing-inspired ribbed actuator composed of a tilted bilayer with distinct alignment directions is shown in Supplementary Fig. 9b and Supplementary Movie 7. The non-parallel configuration relative to the substrate enables deformation at programmable angles, highlighting its potential for diverse applications. Furthermore, these 4D-microprinted microactuators exhibit sufficient force and mechanical robustness to perform functional tasks at the microscale. A stag beetle-inspired LCN-based microgripper further highlights the versatility of this approach. Its two mandibles incorporate dual-aligned micropillars, enabling controlled outward bending under light stimulation (Fig. 6a), thereby allowing rapid and precise light-driven actuation for object manipulation (Supplementary Fig. 6d-f). The functionality of this 4D-microprinted device is further demonstrated by the secure capture and release of a 50 μm microsphere onto a designated platform (Fig. 6b, Supplementary Movie 8). Unlike conventional LCN microgrippers and microhands, which require intricate pre-alignment layers, micro-cell replica molding^52^, or laborious post-assembly micromanipulation^53^, our platform enables one-step, in situ fabrication in which alignment and actuation functionality are programmed directly during DLW, without manual post-processing. We further demonstrate a scalable, application-oriented self-activated LCN microgripper integrated on a cell-culture cavity slide, providing a working clearance (cell gap) of ~700 μm. Using DLW with an air objective, we print a larger-voxel architecture with *dz* = 10 μm and a 0.5 μm hatch spacing (72 mW, 1200 μm/s), yielding a gripper of ~160 μm × 60 μm × 600 μm (Fig. 6c). The device exhibits target-triggered autonomous grasping. A bottom-up fiber laser (515 nm, 113 mW) is aligned through the gap between the two pillars, and closure is triggered only when a highly scattering target enters the gap, thereby enhancing local photothermal heating through target-induced scattering. In contrast, transparent or weakly scattering targets do not trigger actuation. Turning the laser off reopens the grippers and releases the target, enabling a directly translatable route to event-driven micromanipulation for microfluidics and cell handling (Fig. 6d and Supplementary Movie 9).

In summary, we have developed a robust diffusion-guided alignment strategy for TPP, enabling volumetric 3D printing of LCN microstructures with spatially programmable anisotropy. This strategy establishes a self-regulating feedback mechanism, in which the morphology of each printed layer governs the mesogen orientation of the next, allowing seamless integration of multiple alignment domains within fully volumetric 3D architectures. The technique enables complex, reversible out-of-plane deformations that are unattainable using conventional planar alignment techniques, thereby expanding the design space for functional microsystems. Leveraging this capability, we demonstrate a suite of mechanically robust, task-capable microactuators, including a stag beetle-inspired microgripper capable of capturing, transporting, and releasing 50 μm microspheres. These demonstrations underscore that our approach not only achieves precise structural programmability but also delivers sufficient mechanical strength to execute real tasks at the microscale.

Conceptually, this mechanism parallels anisotropic wall thickening in plant morphogenesis, where each newly formed layer continuously redefines the local environment and thus the alignment of the LCN precursor mixture, guiding the growth of subsequent layers. This approach establishes a synthetic design principle for hierarchical 3D structure formation, capable of mimicking morphogenetic processes observed in nature. The anisotropic stacking strategy reflects the morphogenetic logic of natural tissues, where the orientation and progression of growth zones define complex 3D form^54^. Translating this concept into materials design, 3D anisotropy is achieved through a continuous stacking sequence that follows the evolving surface profile, while diffusion-guided alignment adapts dynamically to the changing local environment throughout the growing microstructure. Moreover, this strategy is potentially applicable to other material systems, including soft polymers, responsive hydrogels, and hybrid nanocomposites. Its intrinsic microscale precision makes it especially suited for applications in microfluidic platforms, where integrated actuation can revolutionize functionality. By mimicking hierarchical natural processes, our platform also serves as a versatile model for studying mechanical behaviors across scales, from the cellular level to the tissue scale in both biological and plant systems. This capability opens promising avenues for investigating cellular mechanics, tissue morphogenesis, and the mechanobiology of plant tissues, thereby broadening the impact of our approach well beyond conventional 3D alignment technology in TPP.

## Methods

### LCN precursors for TPP

The mesogenic monomer 4-((6-(Acryloyloxy)hexyl)oxy)phenyl 4-methoxybenzoate and the crosslinker 1,4-bis[4-(3-acryloyloxypropyloxy)benzoyloxy]-2-methylbenzene (RM257) were purchased from Synthon Chemicals. The photoinitiator Irgacure 369 was purchased from Sigma Aldrich. All the materials were used as received. The formulation of the LCN precursor was 66 wt% of LC monomers, 33 wt% of crosslinker RM257 and 1 wt% of initiator Irgacure 369. The mixture was heated and infiltrated into a LC cell at 80 °C to ensure homogenous filling. The cell consisted of two thin cover glasses (0.17 mm thick) spaced by 50 μm spacers at the edges. While pure LC monomers typically exhibited isotropic orientation on common untreated glass substrates, our LCN precursor mixture exhibited homeotropic alignment. This behavior is attributed to the relatively hydrophobic nature of the thin cover glass surfaces in contact with the LCN precursor, which promoted vertical alignment of the LC mesogens without the need for surface treatment.

### Fabrication of TPP-based microstructures

3D microstructures were fabricated using a Laser Nanofactory workstation system (Femtika), operating at a central wavelength of 780 nm, with a pulse duration of <100 fs and a repetition rate of 100 MHz. An oil-immersion objective lens (Plan-Apochromat 63×/1.4 Oil M27, Zeiss) was employed to tightly focus the laser beam into the LCN precursor. The writing speed was set to 2200 μm/s, and the laser power was maintained at 16 mW to ensure high-resolution, high-fidelity fabrication. Following laser exposure, the samples were developed in a 1:1 mixture of n-propanol and isopropanol at 60 °C for ~30 min to remove residual unpolymerized LC monomers. After removing the cover glass, the 3D microstructures were clearly revealed with excellent structural integrity. To eliminate the remaining development solution, the samples were immersed in deionized (DI) water for 5 min.

### Post-treatment for photothermal actuation

For photothermal actuation, an azo-dye solution was delivered using a micropipette to deposit a droplet directly onto the microstructures after development. The additive was prepared by dissolving powdered Disperse Red 1 (purchased from Sigma-Aldrich) in butyl acrylate (BA, 99%; purchased from TCI, Tokyo, Japan). The resulting solution was filtered through a hydrophilic PTFE syringe filter (0.22 μm pore size) to remove any undissolved dye particles. A controlled volume of 15 μL of the filtered solution was carefully dispensed onto the pre-fabricated 3D LCN-based microactuators using a micropipette. The samples were then thermally treated at 50 °C for 12 min. During this process, dye infiltration occurred within the already-formed LCN matrix, which enabled a localized photothermal response without compromising the structural integrity or orientational fidelity of the written microactuator.

### Programmable writing path for 3D printing of LCN-based microstructures

The design and fabrication of LCN-based microstructures were carried out using a programmable laser writing path strategy. Structural geometries were first defined in 3D and then decomposed into two-dimensional layers, which were further segmented into one-dimensional line elements for layer-by-layer fabrication. The start and end coordinates of each line segment were extracted and compiled using 3DPoli Compiler software (Femtika), which converted the geometric data into machine-readable commands to control both the scanning direction and spatial positioning of the femtosecond laser. This approach enabled precise spatial control of the laser focus during the TPP process, facilitating the construction of complex 3D micro- and nanostructures with programmable alignment and geometry.

### Optical characterization and deformation analysis

Optical images and real-time motion tracking were conducted using an optical microscope (Olympus BX53-P). Birefringence analysis was performed under cross-polarized light with the insertion of a full-wave plate (550 nm, Olympus U-GAN) to evaluate LC alignment within the structures. Side-view observations of deformation were captured using a custom-built optical microscope positioned at a tilted angle to visualize out-of-plane actuation. The morphology of the fabricated 3D LCN-based microstructures was characterized using a Field Emission Scanning Electron Microscope (FE-SEM, JSM-7610F, JEOL). To avoid damaging the polymeric network, the imaging was performed at a low accelerating voltage of 10 kV. Thermally induced deformation of LCN-based microstructures was measured using a custom-designed heating stage mounted on the microscope platform, enabling in situ observation during temperature variation. The displacement measurements were subsequently analyzed using ImageJ software.

## Supplementary information

Supplementary Information

Description of Additional Supplementary Files

Supplementary Movie 1

Supplementary Movie 2

Supplementary Movie 3

Supplementary Movie 4

Supplementary Movie 5

Supplementary Movie 6

Supplementary Movie 7

Supplementary Movie 8

Supplementary Movie 9

Transparent Peer Review file

## Source data

Source Data

## Data Availability

All data are available from the corresponding author upon request. The raw data underlying the results presented in the main text and Supplementary Information have been deposited in the Zenodo database at 10.5281/zenodo.19413139. Source data are provided with this paper.

## References

[1] Hines, L., Petersen, K., Lum, G. Z. & Sitti, M. Soft actuators for small-scale robotics. *Adv. Mater.* **29**, 1603483 (2017). 10.1002/adma.201603483 28032926

[2] Reynolds, M. F. et al. Microscopic robots with onboard digital control. *Sci. Robot.* **7**, eabq0612 (2022). 10.1126/scirobotics.abq2296 36129993

[3] Miskin, M. Z. et al. Electronically integrated, mass-manufactured, microscopic robots. *Nature* **584**, 557–561 (2020). 32848225 10.1038/s41586-020-2626-9

[4] Mol, G. N., Harris, K. D., Bastiaansen, C. W. M. & Broer, D. J. Thermo-mechanical responses of liquid-crystal networks with a splayed molecular organization. *Adv. Funct. Mater.* **15**, 1155–1159 (2005).

[5] White, T. J. & Broer, D. J. Programmable and adaptive mechanics with liquid crystal polymer networks and elastomers. *Nat. Mater.* **14**, 1087–1098 (2015). 26490216 10.1038/nmat4433

[6] Ge, F. & Zhao, Y. Microstructured actuation of liquid crystal polymer networks. *Adv. Funct. Mater.* **29**, 1901890 (2019).

[7] Sharma, A. et al. Biocompatible, biodegradable and porous liquid crystal elastomer scaffolds for spatial cell cultures. *Macromol. Biosci.* **15**, 200–214 (2015). 25303674 10.1002/mabi.201400325

[8] Martella, D. et al. Liquid crystalline networks toward regenerative medicine and tissue repair. *Small* **13**, 1702677 (2017). 10.1002/smll.201702677 29045016

[9] Yasa, I. C., Tabak, A. F., Yasa, O., Ceylan, H. & Sitti, M. 3D-printed microrobotic transporters with recapitulated stem cell niche for programmable and active cell delivery. *Adv. Funct. Mater.* **29**, 1808992 (2019).

[10] Nemati, Y. et al. Magneto-photochemically responsive liquid crystal elastomer for underwater actuation. *ACS Appl. Mater. Interfaces* **17**, 5316–5325 (2025). 39788547 10.1021/acsami.4c14704 PMC11758782

[11] Zhang, J. et al. Liquid crystal elastomer-based magnetic composite films for reconfigurable shape-morphing soft miniature machines. *Adv. Mater.* **33**, 2006191 (2021). 33448077 10.1002/adma.202006191 PMC7610459

[12] Zheng, C. et al. Light-driven micron-scale 3D hydrogel actuator produced by two-photon polymerization microfabrication. *Sens. Actuators B Chem.* **304**, 127345 (2020).

[13] Van Oosten, C. L., Harris, K. D., Bastiaansen, C. W. M. & Broer, D. J. Glassy photomechanical liquid-crystal network actuators for microscale devices. *Eur. Phys. J. E* **23**, 329–336 (2007). 17687511 10.1140/epje/i2007-10196-1

[14] White, T. J. et al. A high frequency photodriven polymer oscillator. *Soft Matter* **4**, 1796–1798 (2008).

[15] Zeng, H., Wani, O. M., Wasylczyk, P., Kaczmarek, R. & Priimagi, A. Self-regulating iris based on light-actuated liquid crystal elastomer. *Adv. Mater.* **29**, 1701814 (2017). 10.1002/adma.201701814 28589679

[16] Rogóż, M., Zeng, H., Xuan, C., Wiersma, D. S. & Wasylczyk, P. Light-driven soft robot mimics caterpillar locomotion in natural scale. *Adv. Opt. Mater.* **4**, 1689–1694 (2016).

[17] Cheng, M. et al. Light-fueled polymer film capable of directional crawling, friction-controlled climbing, and self-sustained motion on a human hair. *Adv. Sci.* **9**, 2103090 (2022). 10.1002/advs.202103090 PMC8728837 34713627

[18] Yang, J., Zhang, H., Berdin, A., Hu, W. & Zeng, H. Dandelion-inspired, wind-dispersed polymer-assembly controlled by light. *Adv. Sci.* **10**, 2206752 (2022). 10.1002/advs.202206752 PMC9982548 36574479

[19] Zhu, C. et al. Light-driven liquid crystal elastomer actuators based on surface plasmon resonance for soft robots. *ACS Appl. Mater. Interfaces* **16**, 69858–69869 (2024). 39636093 10.1021/acsami.4c14718

[20] Ahn, C., Liang, X. & Cai, S. Bioinspired design of light-powered crawling, squeezing, and jumping untethered soft robot. *Adv. Mater. Technol.* **4**, 1900185 (2019).

[21] Shahsavan, H. et al. Bioinspired underwater locomotion of light-driven liquid crystal gels. *Proc. Natl. Acad. Sci. USA* **117**, 5125–5133 (2020). 32094173 10.1073/pnas.1917952117 PMC7071923

[22] Wie, J. J., Shankar, M. R. & White, T. J. Photomotility of polymers. *Nat. Commun.* **7**, 13260 (2016). 27830707 10.1038/ncomms13260 PMC5109552

[23] Cheng, Y.-C., Lu, H.-C., Lee, X., Zeng, H. & Priimagi, A. Kirigami-based light-induced shape-morphing and locomotion. *Adv. Mater.* **32**, 1906233 (2019). 10.1002/adma.201906233 31834665

[24] Palagi, S. et al. Structured light enables biomimetic swimming and versatile locomotion of photoresponsive soft microrobots. *Nat. Mater.* **15**, 647–653 (2016). 26878315 10.1038/nmat4569

[25] Wani, O. M., Zeng, H. & Priimagi, A. A light-driven artificial flytrap. *Nat. Commun.* **8**, 15546 (2017). 28534872 10.1038/ncomms15546 PMC5457518

[26] Pilz da Cunha, M. et al. A soft transporter robot fueled by light. *Adv. Sci.* **7**, 1902842 (2020). 10.1002/advs.201902842 PMC7055549 32154076

[27] Guin, T. et al. Layered liquid crystal elastomer actuators. *Nat. Commun.* **9**, 2531 (2018). 29955053 10.1038/s41467-018-04911-4 PMC6023890

[28] Zeng, X. et al. Reconfigurable liquid crystal elastomer director patterns for multi-mode shape morphing. *Crystals* **14**, 357 (2024).

[29] Ware, T. H., McConney, M. E., Wie, J. J., Tondiglia, V. P. & White, T. J. Voxelated liquid crystal elastomers. *Science* **347**, 982–984 (2015). 25722408 10.1126/science.1261019

[30] Gladman, A. S., Matsumoto, E. A., Nuzzo, R. G., Mahadevan, L. & Lewis, J. A. Biomimetic 4D printing. *Nat. Mater.* **15**, 413–418 (2016). 26808461 10.1038/nmat4544

[31] Kotikian, A., Truby, R. L., Boley, J. W., White, T. J. & Lewis, J. A. 3D printing of liquid crystal elastomeric actuators with spatially programmed nematic order. *Adv. Mater.* **30**, 1706164 (2018). 10.1002/adma.201706164 29334165

[32] Ambulo, C. P. et al. Four-dimensional printing of liquid crystal elastomers. *ACS Appl. Mater. Interfaces* **9**, 37332–37339 (2017). 28967260 10.1021/acsami.7b11851

[33] López-Valdeolivas, M., Liu, D., Broer, D. J. & Sánchez-Somolinos, C. 4D printed actuators with soft-robotic functions. *Macromol. Rapid Commun.* **39**, 1700710 (2018). 10.1002/marc.201700710 29210486

[34] Maruo, S., Nakamura, O. & Kawata, S. Three-dimensional microfabrication with two-photon-absorbed photopolymerization. *Opt. Lett.* **22**, 132–134 (1997). 18183126 10.1364/ol.22.000132

[35] Kawata, S., Sun, H.-B., Tanaka, T. & Takada, K. Finer features for functional microdevices. *Nature* **412**, 697–698 (2001). 11507627 10.1038/35089130

[36] Zeng, H. et al. High-resolution 3D direct laser writing for liquid-crystalline elastomer microstructures. *Adv. Mater.* **26**, 2319–2322 (2014). 24421068 10.1002/adma.201305008

[37] Zeng, H. et al. Light-fueled microscopic walkers. *Adv. Mater.* **27**, 3883–3887 (2015). 26033690 10.1002/adma.201501446 PMC4660875

[38] Chen, L. et al. Development of direct-laser-printable light-powered nanocomposites. *ACS Appl. Mater. Interfaces* **11**, 19541–19553 (2019). 31059220 10.1021/acsami.9b05871

[39] Guo, Y., Zhang, J., Hu, W., Khan, M. T. A. & Sitti, M. Shape-programmable liquid crystal elastomer structures with arbitrary three-dimensional director fields and geometries. *Nat. Commun.* **12**, 5936 (2021). 34642352 10.1038/s41467-021-26136-8 PMC8511085

[40] Guo, Y., Shahsavan, H., Davidson, Z. S. & Sitti, M. Precise control of lyotropic chromonic liquid crystal alignment through surface topography. *ACS Appl. Mater. Interfaces* **11**, 36110–36117 (2019). 31532609 10.1021/acsami.9b12943

[41] Guo, Y., Shahsavan, H. & Sitti, M. 3D microstructures of liquid crystal networks with programmed voxelated director fields. *Adv. Mater.* **32**, 2002753 (2020). 10.1002/adma.202002753 PMC7610484 32767434

[42] Hsu, L.-Y. et al. Alignment and actuation of liquid crystals via 3D confinement and two-photon laser printing. *Sci. Adv.* **10**, eadq2597 (2024). 39241061 10.1126/sciadv.adq2597 PMC11378907

[43] Gulati, L., Sánchez-Somolinos, C., Giesselmann, F. & Fischer, P. Aligning and observing the liquid crystal director in 3D using small magnetic fields and a wedge-cell. *Adv. Funct. Mater.* **35**, 2413513 (2025).

[44] Carlotti, M., Tricinci, O., den Hoed, F., Palagi, S. & Mattoli, V. Direct laser writing of liquid crystal elastomers oriented by a horizontal electric field. *Open Res. Eur.* **1**, 129 (2021). 37645193 10.12688/openreseurope.14135.2 PMC10445945

[45] Münchinger, A. et al. Multi-photon 4D printing of complex liquid crystalline microstructures by in situ alignment using electric fields. *Adv. Mater. Technol.* **7**, 2100944 (2022).

[46] Hisano, K. et al. Scanning wave photopolymerization enables dye-free alignment patterning of liquid crystals. *Sci. Adv.* **3**, e1701610 (2017). 29152567 10.1126/sciadv.1701610 PMC5681215

[47] Hisano, K. et al. Alignment layer-free molecular ordering induced by masked photopolymerization with non-polarized light. *Appl. Phys. Express* **9**, 072601 (2016).

[48] Zhang, Z. et al. 3D directional assembly of liquid crystal molecules. *Adv. Mater.* **36**, 2401533 (2024). 10.1002/adma.202401533 38794830

[49] Ritacco, T., Mazzulla, A., Giocondo, M., Cipparrone, G. & Pagliusi, P. In situ control of reactive mesogens alignment during 3D printing by two-photon lithography. *Adv. Sci.* **12**, 2415159 (2025). 10.1002/advs.202415159 PMC12140314 40162487

[50] Schmidt, S. T. *Transmitted Light Microscopy of Rock-Forming Minerals: An Introduction to Optical Mineralogy*, Ch. 5 (Springer Nature, 2023).

[51] Donato, S. et al. Liquid crystalline network microstructures for stimuli responsive labels with multi-level encryption. *Small* **19**, 2300828 (2023). 10.1002/smll.202306802 38063817

[52] Potekhina, A. & Wang, C. H. Liquid crystal elastomer based thermal microactuators and photothermal microgrippers using lateral bending beams. *Adv. Mater. Technol.* **7**, 2101732 (2022).

[53] Martella, D., Nocentini, S., Nuzhdin, D., Parmeggiani, C. & Wiersma, D. S. Photonic microhand with autonomous action. *Adv. Mater.* **29**, 1704047 (2017). 10.1002/adma.201704047 28976033

[54] Coen, E. & Cosgrove, D. J. The mechanics of plant morphogenesis. *Science* **379**, eade8055 (2023). 36730409 10.1126/science.ade8055

