# Supplementary material for: Diffusion-guided 4D microprinting of soft microactuators
Source: Nat Commun. 2026 May 14;17:6427. doi: 10.1038/s41467-026-73035-x (PMC13376197; doi:10.1038/s41467-026-73035-x)
Supplement: Supplementary file 1 — Supplementary Information [file 41467_2026_73035_MOESM1_ESM.pdf]

Supplementary information

Diffusion-guided 4D microprinting of soft microactuators

Wei-Ting Hsu[1], Po-An Tsou[1], Hsin-Jung Chou[1], Tsung-Kai Lin[1] & Yu-Chieh Cheng[1]*

[1]Department of Electro-Optical Engineering, National Taipei University of Technology (Taipei Tech) No. 1, Sec. 3, Zhongxiao East Road, Da'an District, Taipei 10608, Taiwan (R.O.C.)

*email: yu-chieh.cheng@mail.ntut.edu.tw

Table of contents:

Supplementary Notes 1-10:

Supplementary Note 1. Sample Preparation, TPP Fabrication, and Experimental Setup.
Supplementary Note 2. Quantitative analysis of optical path difference for single-layer LCNs.
Supplementary Note 3. Role of Inter-Layer Delay in Mesogen Diffusion during Bulk LCN Fabrication.
Supplementary Note 4. 4D microprinting of dual-alignment LCN-based microstructures.
Supplementary Note 5. Microprinting of locally programmable in-plane LCN microstructures.
Supplementary Note 6. Ultrafast Actuation and Kinematic Analysis of Light-Driven Micro-actuators.
Supplementary Note 7. Thermomechanical cycling and repeatability of the suspended microstrip.
Supplementary Note 8. Mechanism of Diffusion-Guided Alignment.
Supplementary Note 9. Triangular stacking-enabled programmed twist.
Supplementary Note 10. 3D stacking on non-planar substrates.

Supplementary Figures 1-9:

Supplementary Figure. 1 | Preparation of samples for diffusion-driven alignment observation and light-driven shaping of TPP-fabricated LCN microactuators.
Supplementary Figure. 2 | Quantitative OPD analysis for single-layer LCNs.
Supplementary Figure. 3 | Interference color observation for uniform molecular orientation in LCN-based microstructures.
Supplementary Figure. 4 | Design and thermally responsive behavior of dual-alignment LCN-based microstructures.
Supplementary Figure. 5 | Locally programmed in-plane orientation in a continuous honeycomb lattice.
Supplementary Figure. 6 | Photothermal actuation metrics under laser excitation: suspended microstrip shrinkage and stag beetle-inspired microgripper jaw opening.
Supplementary Figure. 7 | Repeatable thermal shrinkage actuation of the suspended microstrip.
Supplementary Figure. 8 | Diffusion-guided voxel scanning and layer stacking enable programmed twisting actuation.
Supplementary Figure. 9 | Biomimetic light-responsive 3D/4D LCN-based microactuators.

Supplementary Table 1:

Supplementary Table 1 | Fabrication parameters, OPD, and calculated LC director tilt angles θ relative to the z-axis for dual-aligned LCN micropillars (A-H).

Supplementary References: 1-2

Supplementary Note 1. Sample Preparation, TPP Fabrication, and Experimental Setup.

The LC precursor mixture was introduced into a capillary cell at 80 °C and cooled to 45 °C to reach the nematic phase. After 20 min of thermal equilibration, microstructures were fabricated via two-photon polymerization (TPP) using a high-NA (63× oil immersion) objective, as illustrated in Supplementary Fig. 1a. During direct laser writing (DLW), voxel-level scanning was performed following the predesigned 3D model with a scanning speed of 2200 μm s^{-1} and laser power of 16 mW. The resulting voxel dimensions were measured to be approximately 0.45 μm in width (w) and 1.5 μm in height (h), enabling high-resolution 3D structuring within the liquid crystal network (LCN).
For alignment analysis, the liquid crystal (LC) cell containing the TPP-fabricated microstructures and unpolymerized precursors was uniformly UV-cured (365 nm, 45 °C, 5 min) to fix the orientation of diffused mesogens along the polymer edges, as shown in Supplementary Fig. 1b. Birefringence color shifts were then characterized using a POM setup with crossed polarizers and a 550 nm full-wave plate to resolve the LC director fields. After inserting a full-wave retarder, the phase retardance introduces wavelength-dependent polarization changes, resulting in distinct color variations. As shown in Supplementary Fig. 1c, the birefringence-induced color shifts reveal the orientation of the LC directors: regions with vertical alignment shift from black to purple, while those with -45° horizontal and +45° horizontal alignments change from white to blue and yellow-orange, respectively, when the slow axis of the full-wave plate is set at −45°. To ensure reliable visualization, UV post-curing was required because OM illumination contains UV components that could otherwise cause unintended and inconsistent polymerization.

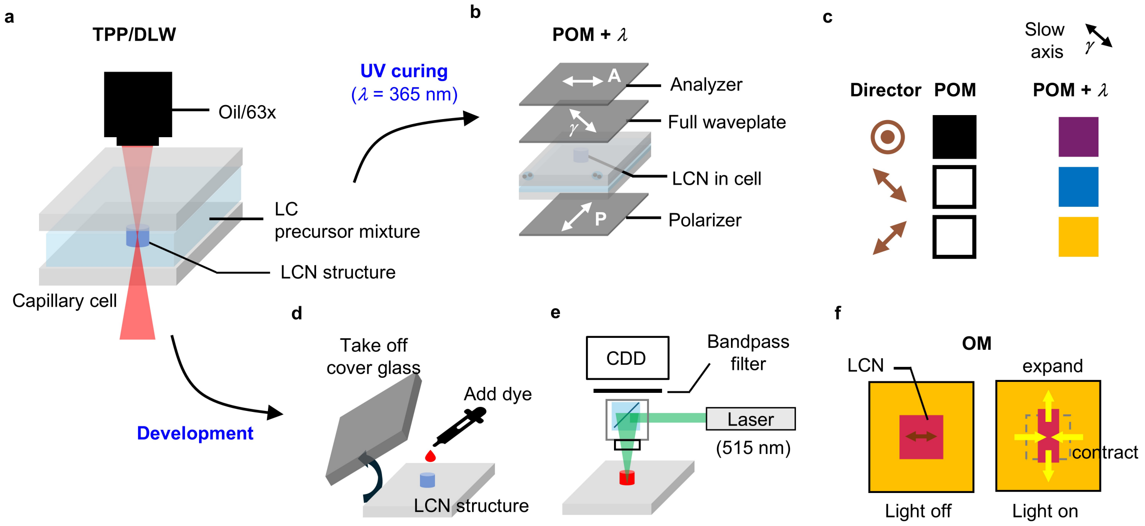

Supplementary Figure. 1 | Preparation of samples for diffusion-driven alignment observation and light-driven shaping of TPP-fabricated LCN microactuators. a Schematic showing the TPP fabrication in a LC cell, followed by observation under polarized optical microscope (POM). **b** After UV curing, the sample is placed under a POM + λ system consisting of a polarizer (P) and an analyzer (A), with a 550 nm full-wave plate inserted between the crossed polarizers. **c** Schematic illustration of the LC director orientations and their corresponding birefringence colors observed under POM and POM + λ (with a 550 nm full-wave plate inserted). The slow axis of the plate is set at γ = −45°. **d** After the sample is developed to remove unpolymerized material, the cover glass is carefully removed (gray plate being lifted). A red droplet represents the Disperse Red 1 dye solution being applied to the LCN microstructure (blue solid object) to impart light-induced responsiveness. **e** A 515 nm laser is coupled into the optical microscope via a beamsplitter, allowing real-time visualization of photothermal actuation of LCN microstructures. **f** The central raspberry-red region corresponds to the LCN microstructure, where the director orientation ($\mathbf{n}$) is aligned along the

horizontal axis (indicated by the brown arrow). The black arrows illustrate the deformation directions during photothermal actuation, as observed under optical microscope (OM).

For photothermal actuation, unpolymerized material was removed in a 1:1 mixture of n-propanol and isopropanol, and the cover glass was carefully detached to expose freestanding structures. A 15 μL droplet of Disperse Red 1 dye, dissolved in butyl acrylate, was applied to the fabricated structures, enabling photothermal deformation (Supplementary Fig. 1d). For dynamic actuation studies, a 515 nm excitation laser was introduced laterally through a beamsplitter integrated into the optical microscope, enabling real-time monitoring of photothermal deformation during imaging, as illustrated in Supplementary Fig. 1e. To prevent potential damage to the CCD, a bandpass filter was placed in front of the detector to block wavelengths below 532 nm. As a result, the background of the OM image appears yellow due to the effect of the filters, as shown in Supplementary Fig. 1f, which enables clear observation of light-driven actuation under the microscope. Upon laser illumination, photothermal heating induces anisotropic contraction along the director and expansion perpendicular to it, resulting in the observed deformation.

Supplementary Note 2. Quantitative analysis of optical path difference for single-layer LCNs.

Supplementary Fig. 2 quantifies how lateral size (20, 40, 100 μm) influences mesogen orientation within single-layer LCNs. For small lateral sizes (e.g., 20 μm, short inter-line delays), diffusion is insufficient, and the mesogens remain predominantly vertical, as shown in Supplementary Fig. 2a. The inter-line delay time (dt) is calculated by summing the time required for voxel scanning along each line and the stage movement between adjacent lines:

$$\mathrm{dt} = \frac{L}{V_{\mathrm{scan}}} + \frac{L}{V_{\mathrm{move}}}. \tag{1}$$

For 20, 40 and 100 μm-long single-layer LCNs, with V_{scan}= 2200 μm /s and V_{move}= 500 μm /s, the calculated inter-line delay is approximately 49 ms, 98 ms and 245 ms, respectively.

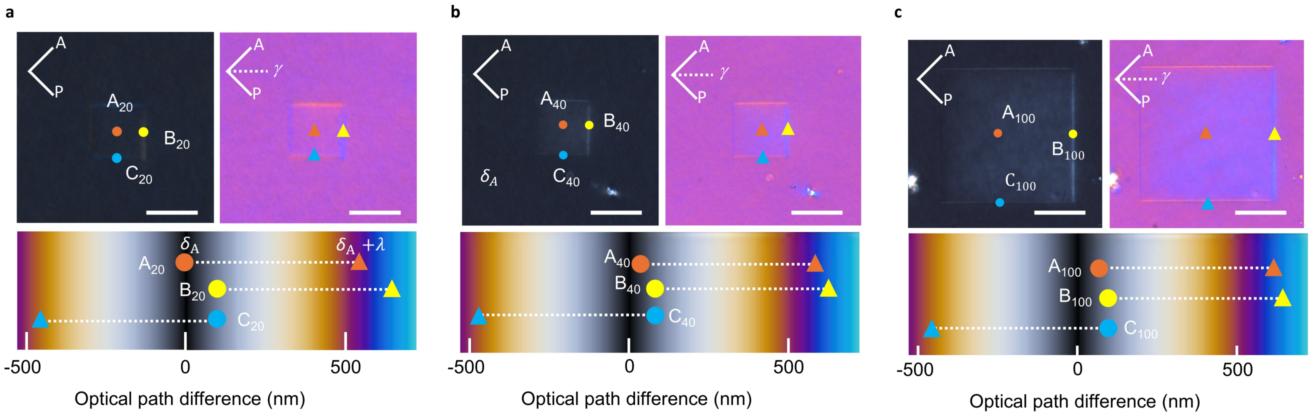

Supplementary Figure. 2 | Quantitative OPD analysis for single-layer LCNs. POM and POM + λ images of single-layer LCNs with lateral sizes of 20 μm (a), 40 μm (b), and 100 μm (c), showing birefringence responses at three representative regions for each structure: point A (orange, center of the LCN layer), point B (yellow, right edge), and point C (cyan, bottom edge). The measured colors under POM and POM + λ are mapped onto the birefringence color bar. The dashed line (γ) indicates the slow axis direction of the λ-plate, which provides a retardation of 550 nm, and A and P denote the analyzer and polarizer, respectively. Scale bars: a = 20 μm; b,c = 40 μm.

At point A_{20} in Supplementary Fig. 2a, the POM image exhibits a dark region corresponding to optical path differences (OPD) ≈ 0 nm, confirming vertical alignment consistent with the pretreated anchoring. Under POM with a full-wave retarder (λ = 550 nm), both regions appear purple, indicating an optical retardation of ~550 nm and further confirming vertical alignment. At the right edge (point B_{20}), the POM image shows gray without the retarder and blue with the retarder, corresponding to an optical retardation of ~ 550 nm and indicating mesogen reorientation toward the in-plane direction parallel to the slow axis of the retarder. At the lower edge (point C_{20}), a yellow color shift is observed under POM + λ at the bottom edge, indicating that the OPD under POM + λ is less than 550 nm compared to POM alone. This subtractive retardation confirms that the LC alignment at the bottom edge is perpendicular to the slow axis of the retarder. Together, the observations at points B and C confirm that mesogens adjacent to the voxel sidewalls reorient perpendicular to the LCN edges.

For larger lateral sizes (40 μm and 100 μm), the extended inter-line delay allows sufficient mesogen diffusion into the voxel sidewalls during DLW, resulting in uniform in-plane alignment across both the center and edges of the LCN layer, as shown in Supplementary Fig. 2c. For a 40 μm-long single-layer LCN (top), the shorter inter-line delay (dt ≈ 98 ms) provides insufficient time for complete mesogen diffusion, resulting in a partially tilted alignment with a director tilt angle θ relative to the z-axis. In contrast, for a 100 μm-long single-layer LCN (bottom), the longer inter-line delay (dt ≈ 245 ms) allows sufficient diffusion of unpolymerized mesogens, leading to a uniform in-plane alignment as shown in Supplementary Fig. 2d.

Assuming that the effective thickness of the LCN layer is approximately half of the voxel height (h ≈ 1.5 μm), the theoretical maximum $\mathrm{OPD_{max}}$ for a single LCN layer is given by Eq. (2):

$$\mathrm{OPD_{max}} = \Delta n \cdot \frac{h}{2} \tag{2}$$

where $\Delta n = 0.1375$, yielding $\delta_{\max}$ ≈ 103 nm. An OPD of approximately 100 nm corresponds to a light-gray interference color, which is consistent with the light-gray birefringence observed in the 100 μm-long LCN layer. Therefore, at least an inter-line delay of ≈ 245 ms is required to achieve fully developed in-plane alignment, underscoring that diffusion dominates mesogen orientation at the voxel level.

Supplementary Note 3. Role of Inter-Layer Delay in Mesogen Diffusion during Bulk LCN Fabrication.

3.1 Description and Methodology

Taking the dual-aligned micropillars shown in Supplementary Fig. 3a as an example, the left region (green) and right region (pink) are designed with vertical and horizontal molecular alignments, respectively, where the layer-stacking directions are oriented along the z-axis and x-axis. The resulting alignment depends on whether diffusion is fully completed, which is strongly influenced by the degree of crosslinking within the polymer network. A strongly crosslinked network produces a larger concentration gradient and faster diffusion rates, but limits the penetration depth of diffusing mesogens. Several fabrication parameters, including scanning speed, laser power, and voxel overlap, directly affect the extent of crosslinking and thus determine the required diffusion time.

For effective molecular alignment, the diffusion must be completed within the inter-layer delay (dt), which corresponds to the time required to complete the sequential line-by-line voxel scanning for a single layer before stacking the next layer. The subsequent layer-by-layer stacking then governs the final LC orientation through a diffusion-guided alignment process.

Unlike single-layer LCNs (Supplementary Fig. 2), where the available diffusion time is determined by the inter-line delay between adjacent scanning paths, bulk LCN fabrication is primarily governed by the inter-layer delay—the time

interval before the voxel returns to polymerize an overlapping region from the subsequent layer above. The inter-layer delay time (dt)for each micropillar is calculated based on the fabrication parameters, including voxel line length (L), lateral step size (dx), scanning speed (V_{scan}), and moving speed (V_{move}), according to Eq. (3):

$$\mathrm{dt} = \left(\frac{L}{V_{\mathrm{scan}}}\right) \cdot N_{\mathrm{scan}} + \left(\frac{L}{V_{\mathrm{move}}}\right) \cdot (N_{\mathrm{scan}} - 1) \tag{3}$$

where L = 20 μm is the line length, V_{scan} = 2200 μm /s is the voxel scanning speed, V_{move} = 500 μm /s is the moving speed between scan lines. The number of voxel-line scans per layer, N_{scan} , is calculated by Eq. (4):

$$N_{\mathrm{scan}} = \frac{T}{\mathrm{dz}} + 1 \tag{4}$$

where T is the total layer thickness. A layer step size of dz = 0.5 μm was used for these LCN-based microstructures.

3.2 Experimental Verification via POM

To experimentally verify the molecular alignment, birefringence colors observed under POM were analyzed and mapped onto the interference color bar to determine the corresponding OPD, as shown in Supplementary Fig. 3c. The fabrication parameters, observed birefringence colors, and calculated alignment tilt angles with respect to the z-axis for samples A-H are summarized in Supplementary Table 1.

The OPD measured from the interference color under POM is related to the effective birefringence by:

$$\mathrm{OPD} = \Delta n_{\mathrm{eff}}(\theta) \cdot T \tag{5}$$

where T is the micropillar thickness.

3.3 Calculation of LC Director Tilt Angle

For a uniaxial birefringent medium, the effective extraordinary refractive index $\Delta n_{\mathrm{eff}}(\theta)$depends on the director tilt angle θ with respect to the light propagation direction. Under normal incidence, the propagation direction is along the z-axis ($\boldsymbol{k} \| z$), and θ is defined as the angle between the director (optic axis) and z. The starting point is the index ellipsoid relation:

$$\frac{1}{n_{\mathrm{eff}}(\theta)} = \frac{\cos^2(\theta)}{n_0^2} + \frac{\sin^2(\theta)}{n_e^2} \tag{6}$$

where n_e and n_0 are the extraordinary and ordinary refractive indices, respectively.

Assuming $|\Delta n| \ll n_0$ and defining $\Delta n = n_e - n_0$, we approximate:

$$\frac{1}{n_e^2} = \frac{1}{(\Delta n + n_0)^2} = \frac{1}{n_0^2} \frac{1}{(1 + \frac{\Delta n}{n_0})^2} = \frac{1}{n_0^2}\left(1 - \frac{2\Delta n}{n_0}\right) \tag{7}$$

Substituting into Eq. (6):

$$\frac{1}{n_{\text{eff}}^2(\theta)} = \frac{1}{n_0^2}\left(1 - \frac{2\Delta n}{n_0}\sin^2(\theta)\right) \tag{8}$$

Taking the reciprocal square root and applying $y \ll 1$ applying $(1-y)^{-1/2} \approx 1 + \frac{y}{2}$ yields:

$$n_{\text{eff}}(\theta) \approx n_0 + \Delta n \cdot \sin^2\theta \tag{9}$$

Thus, the effective birefringence is:

$$\Delta n_{\text{eff}}(\theta) \approx \Delta n \cdot \sin^2\theta \tag{10}$$

and Substituting Eq. (10) into Eq. (5), the tilt angle θ can be calculated as:

$$\theta = \sin^{-1}\sqrt{\frac{\text{OPD}}{\Delta n \cdot T}} \tag{11}$$

Thus, the birefringence colors observed under POM are used to determine OPD, which is then combined with the known Δn and structure thickness T to quantitatively extract the LC director tilt angle θ.

The observed interference colors and the corresponding OPD results for the microstructures fabricated with lateral stacking for in-plane alignment are presented in Supplementary Fig. 3. To ensure the accuracy of the extracted optical path difference (OPD) and the derived director tilt angles, the interference colors were validated using the optical compensation method. By alternating the orientation of a full-wave plate's (λ= 550 nm) slow axis relative to the microstructure, we observed the resulting additive and subtractive color shifts. This procedure allowed us to unambiguously determine the specific retardation regime and provide realistic error estimates for the molecular orientation. Consequently, the reported OPD values are not merely based on static visual mapping but are cross-verified through dynamic phase-shift identification, ensuring the reliability of the 3D alignment characterization.

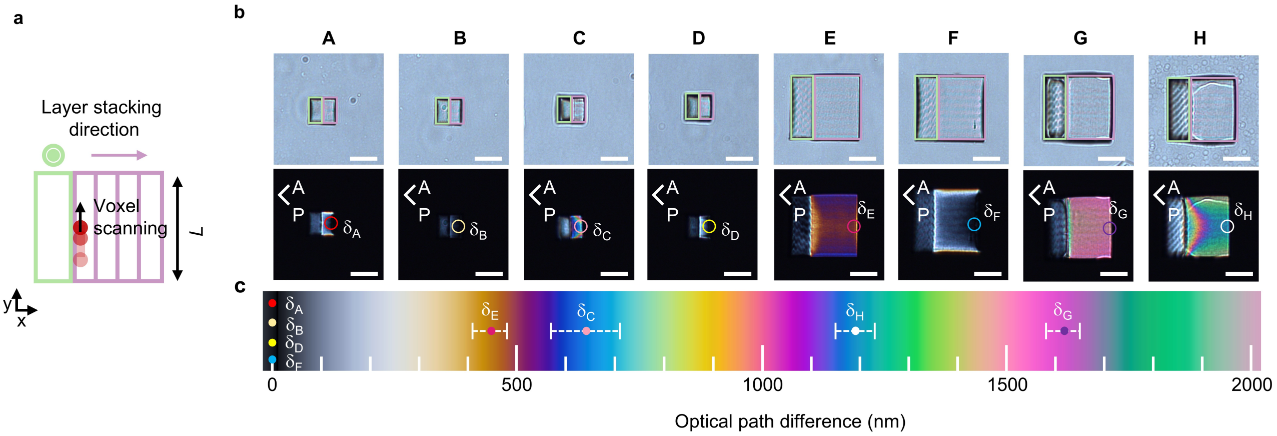

Supplementary Figure. 3 | Interference color observation for uniform molecular orientation in LCN-based microstructures. a Schematic illustration of the voxel scanning strategy and layer stacking direction during DLW fabrication. Each yz-plane layer (indicated by pink lines) is formed by sequential voxel scanning along the y-axis, and the printed layers are constructed in the yz-plane and then stacked along the x-axis. **b** OM (first row) and POM (second row) images show the physical dimensions of the LCN-based microstructures and birefringence colors

induced by the horizontal alignment of the LC director. **c** The estimated OPDs of the layer-stacking regions were derived from birefringence colors observed under POM. The dashed lines indicate the measurement variation range of the OPD for each case. Scale bars: 20 μm. A and P denote the analyzer and polarizer, respectively.

Supplementary Table 1 | Fabrication parameters, OPD, and calculated LC director tilt angles θ relative to the z-axis for dual-aligned LCN micropillars (A-H).

Case	dx (μm)	T (μm)	L (μm)	dt (s)	estimated OPD (nm)	θ (°)	Alignment state
A	0.2	5	20	~0.5	0	0.0	Vertical (dark)
B	0.4	5	20	~0.5	0	0.0	Vertical (dark)
C	0.2	12	20	~1.19	650 nm (range: 590-710 nm)	38.9° (range: 36.7°- 41.0°)	Partial tilt
D	0.4	12	20	~1.19	0	0.0	Vertical (dark)
E	0.2	5	50	~1.25	450 nm (range: 420-480 nm)	54° (range: 51.4°- 56.7°)	Stronger tilt
F	0.4	5	50	~1.25	0	0.0	Vertical (dark)
G	0.2	12	50	~3.0	1610 nm (range: 1590-1630 nm)	81° (range: 79.0°- 83.6°)	Almost in-plane
H	0.4	12	50	~3.0	1190 nm (range: 1150-1230 nm)	58.1° (range: 56.6°- 59.7°)	intermediate tilt

Supplementary Table 1 summarizes the influence of lateral step size (dx) and inter-layer delay (dt) on LC director alignment. At short delays ($\mathrm{dt} \approx$ 0.5 s), the mesogen diffusion time is insufficient, resulting in OPD $= 0$ nm and $\theta = 0°$ for all structures (A, B), indicating vertical alignment. When dt is increased to ~1.19 s and ~1.25 s for thicker structures, partial in-plane reorientation is observed for the smaller step size ($\mathrm{dx} =$ 0.2 μm). In contrast, for the larger step size ($\mathrm{dx} =$ 0.4 μm) under the same dt (D, F), mesogen realignment remains incomplete and OPD ≈ 0, leading to vertical alignment. When dt is further extended to ~3.0 s, in-plane alignment becomes significant for $\mathrm{dx} =$ 0.2 μm: G reaches θ = 81°, showing almost fully in-plane orientation.

This difference originates from how voxel overlap influences both crosslinking density and diffusion driving force. For $\mathrm{dx} =$ 0.2 μm, the large voxel overlap (~0.45 μm voxel size) creates stronger inter-line crosslinking, forming a continuous polymer network and establishing a steeper local concentration gradient of unpolymerized mesogens near the voxel boundaries. These strong gradients drive efficient layer-by-layer diffusion, enabling mesogens to reorient quickly after each printed layer and resulting in faster and more uniform in-plane alignment. In contrast, for $\mathrm{dx} =$ 0.4 μm, the smaller voxel overlap leads to weaker inter-line crosslinking and thus shallower mesogen concentration gradients between adjacent scanning lines. The reduced gradient weakens the diffusion driving force, so it requires longer inter-layer delays to achieve comparable alignment. Even at $\mathrm{dt} \approx$ 3.0 s, the diffusion for $\mathrm{dx} =$ 0.4 μm (H) remains less complete and less uniform compared to $\mathrm{dx} =$ 0.2 μm (G).

Supplementary Note 4. 4D microprinting of dual-alignment LCN-based microstructures.

To demonstrate versatile alignment control, a dual-alignment rectangular micropillar was fabricated by first performing vertical stacking along the z-axis, followed by horizontal stacking along the x-axis. This layer-by-layer stacking strategy facilitates the generation of anisotropic strain across orthogonal domains and enables programmable deformation as schematized in Supplementary Fig. 4a. We further fabricated the dual-aligned rectangular micropillars with dimensions of 12 × 12 × 35 μm and arranged in a radial configuration at 45° intervals (Supplementary Fig. 4b). Upon heating to 220 °C, the micropillars exhibited directional bending toward the center of the array due to thermally induced anisotropic contraction (Supplementary Fig. 4c).

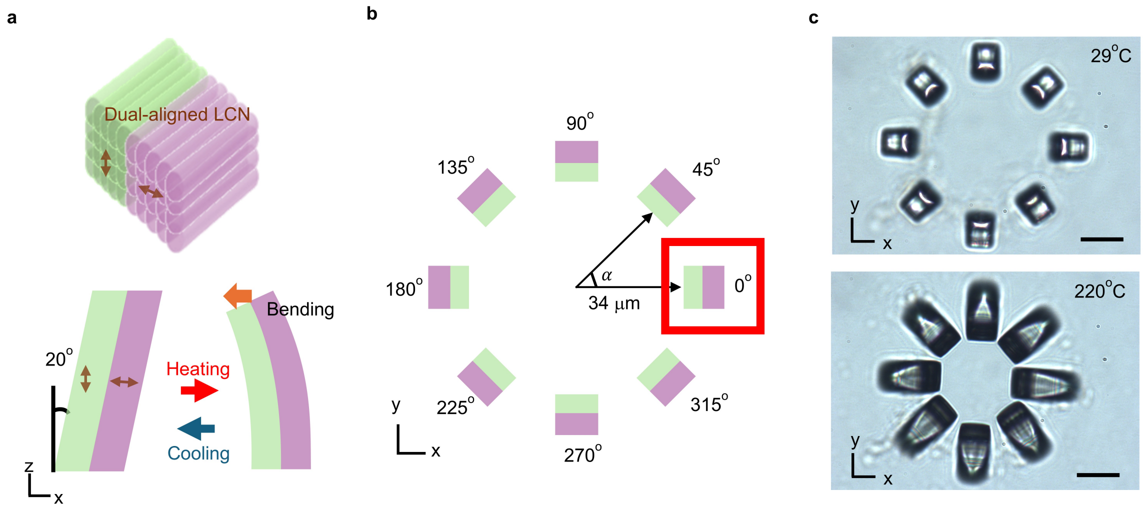

Supplementary Figure. 4 | Design and thermally responsive behavior of dual-alignment LCN-based microstructures. a A dual-aligned micropillar comprising two spatially distinct domains with orthogonal orientations: a vertically aligned (homeotropic) region on the left and a horizontally aligned region on the right (indicated by director field shown in brown). Upon heating, the micropillar bends directionally toward the vertically aligned side due to differential contraction; the deformation is reversible upon cooling. **b** An eight-micropillar LCN-based array is arranged radially, each rotated by 45° (angle α) relative to the x axis. All LCN-based micropillars consist of two orthogonal alignment segments in the xz-plane, with the vertically aligned domain (green rectangle) consistently placed on the inner side. **c** Optical images of an eight-micropillar LCN array and elevated temperature (220 °C), illustrating orientation-dependent thermal bending. All scale bars represent 20 μm.

Supplementary Note 5. Microprinting of locally programmable in-plane LCN microstructures

To clarify the mechanism of in-plane alignment programming, we added a new experiment using a 2 μm-thick cell. By restricting the thickness, we minimize 3D volumetric gradients and isolate the interfacial interaction between the polymerized front and the surrounding precursor. The results show that mesogen ordering is dictated by the diffusion front emanating from the sidewalls of previously polymerized segments, such that the local director aligns perpendicular to the programmed boundary of each hexagon edge and can be prescribed edge by edge (Supplementary Fig. 5). Polarized optical microscopy (POM) and POM with a λ-plate confirm this locally programmed orientation through distinct birefringence contrast and color shifts, demonstrating that multiple in-plane directions can be integrated within a single continuous film. Using the same writing and stacking parameters, we further confirm that this locally programmed orientation is preserved across different honeycomb unit sizes (edge lengths L = 2, 4, and 10

μm); the POM (and POM + λ) images show consistent, edge-dependent birefringence patterns across these scales (Supplementary Fig. 5). This capability enables, for example, spatial control of the retardation slow axis across a continuous LCN layer for advanced optical functionalities.

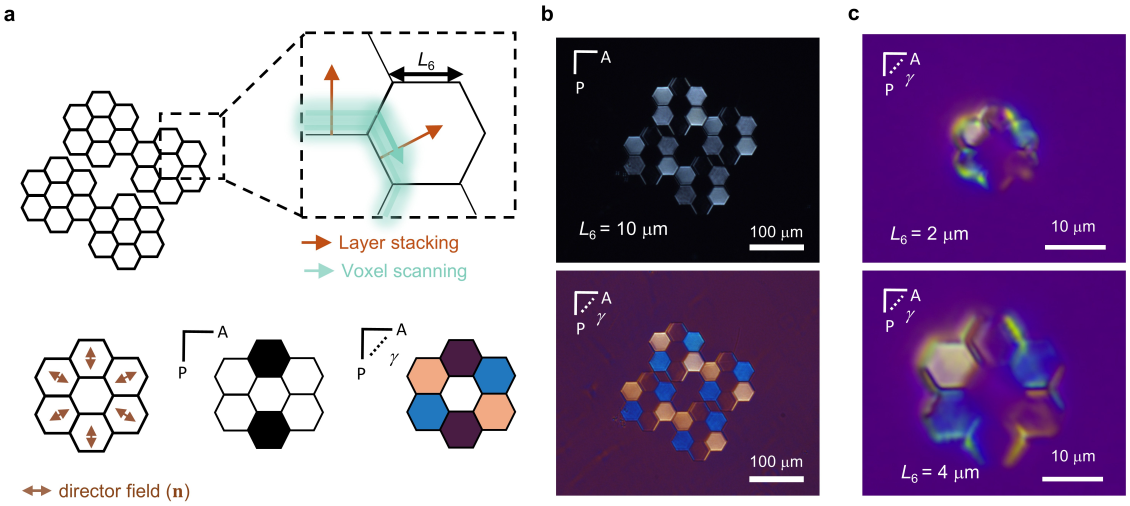

Supplementary Figure. 5 | Locally programmed in-plane orientation in a continuous honeycomb lattice. a Schematic illustrating sequential writing of hexagon-edge segments, where the effective stacking direction (orange arrows) follows each edge and the diffusion front from the previously polymerized segment templates mesogen ordering in the adjacent precursor, thereby programming radially varying in-plane director reorientation from edge to edge. **b** POM and POM + λ images(L_6 = 10 μm) showing spatially varying birefringence contrast/color, confirming locally distinct in-plane director orientations. c POM + λ images of honeycomb lattices with L_6 = 2 μm and 4 μm, demonstrating that locally programmed in-plane liquid crystal director orientations around the hexagonal edges remain resolvable at reduced feature sizes. The dashed line (γ) indicates the slow axis direction of the λ-plate, which provides a retardation of 550 nm, and A and P denote the analyzer and polarizer, respectively.

Supplementary Note 6. Ultrafast Actuation and Kinematic Analysis of Light-Driven Micro-actuators.

To quantitatively evaluate the mechanical response of the micro-soft robots, we first investigated the longitudinal shrinkage of a benchmark pillar structure (Supplementary Fig. 6a). Upon laser irradiation, the micro-pillar exhibited immediate deswelling, with the maximum shrinkage strain reaching approximately 18.5% under a laser power of 8.7 mW. The actuation process followed a power-dependent characteristic as shown in Supplementary Fig. 6b, as the laser power increased from 3.1 mW to 8.7 mW, both the actuation amplitude and the strain rate significantly intensified. Upon cessation of the laser stimulus, the elastic restoration force, coupled with rapid thermal dissipation at the micro-scale, enabled the structure to return to its initial equilibrium state within ~ 80 ms. Furthermore, the angular displacement Φ was employed as the key metric to characterize the joint flexibility. Under laser excitation, the micro-robot's limbs underwent rapid bending, with the maximum bending angle scaling from 16° to 74° as the power increased from 4 mW to 19.2 mW (Supplementary Fig. 6e). The sharp increase in angular displacement of Φ during the first 40 ms suggests a highly efficient photothermal-to-mechanical energy conversion. In the subsequent recovery phase (Supplementary Fig. 6f), the angular displacement rapidly vanished, confirming that the structural deformation is entirely elastic and free of significant hysteresis. The observed ultrafast actuation in an ambient environment stems from the unique scaling laws at the micro-scale. Unlike macroscopic actuators where thermal diffusion is limited by the

bulk volume, the high surface-to-volume ratio of these micro-structures significantly enhances heat dissipation even in air.

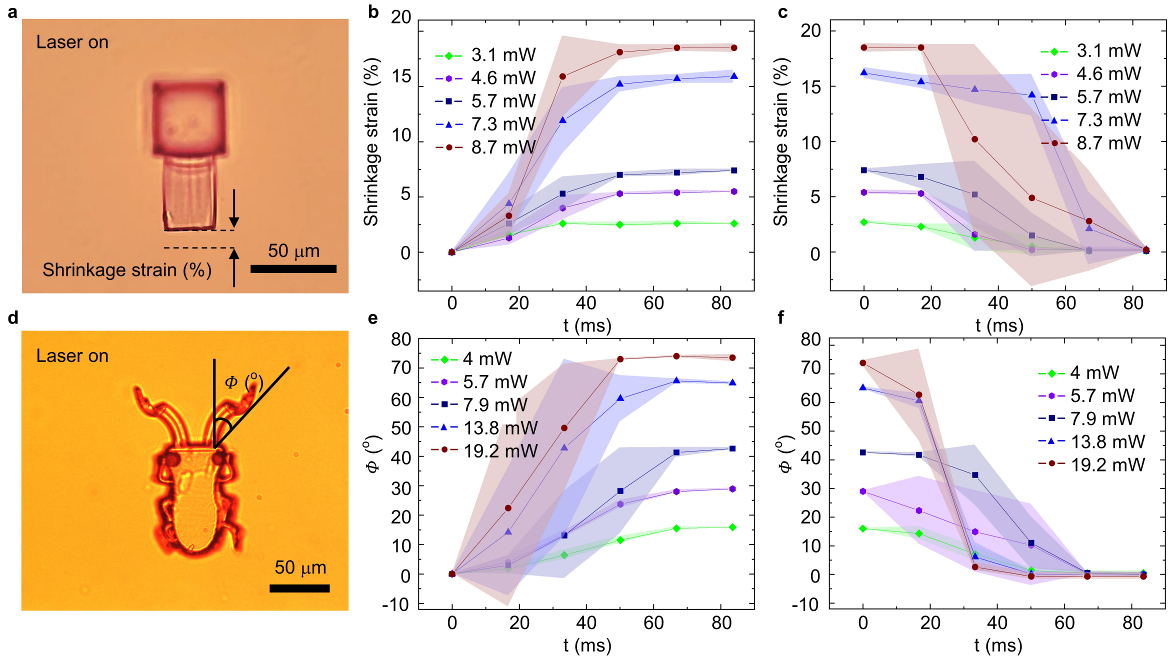

Supplementary Figure. 6 | Photothermal actuation metrics under laser excitation: suspended microstrip shrinkage and stag beetle-inspired microgripper jaw opening.

a Representative optical micrograph of a suspended microstrip under laser excitation (laser on), illustrating the definition of the measured shrinkage strain (%) along the indicated direction (scale bar as indicated). **b** Transient shrinkage strain response following laser turn-on for different excitation powers (color-coded); shaded bands denote variation across repeated measurements/structures (as plotted). **c** Transient recovery of shrinkage strain after laser turn-off for the same power conditions. **d** Representative optical micrograph of a stag beetle-inspired LCN microgripper under laser excitation (laser on), illustrating the definition of the jaw-opening angle Φ (scale bar as indicated). **e** Transient jaw-opening response following laser turn-on for different excitation powers (color-coded); shaded bands denote variation across repeated measurements/structures (as plotted). **f** Transient recovery of the jaw-opening angle after laser turn-off, demonstrating reversible reopening/closing dynamics under photothermal switching. Error bars represent the mean ± standard deviation (n = 3), obtained from repeated measurements of the same sample.

Supplementary Note 7. Thermomechanical cycling and repeatability of the suspended microstrip.

As shown in Supplementary Fig. 7a,b, the thermally driven shrinkage of the suspended microstrips is only weakly dependent on the writing power within our tested range: increasing the writing power results in a slightly smaller peak contraction (within ~1% difference). We attempted to further reduce the writing power to enhance the shrinkage amplitude; however, below the writing power of 16 mW, the printed microstrips became mechanically insufficient during fabrication, failing to maintain structural integrity and thus preventing reliable stacking into complete suspended strips. In principle, diffusion-guided reorientation can still occur at smaller voxel sizes or under reduced laser doses, but capillary forces combined with insufficient network stiffness can trigger mechanical instability, ultimately leading to structural collapse. It should be noted that if the voxel/dose is reduced to the point that polymer

conversion becomes too weak to generate a sufficiently strong local concentration (chemical-potential) gradient, the diffusion-driven influx and subsequent interfacial reorientation will also diminish.

In contrast to the small change in strain amplitude, the activation threshold exhibits a clearer dependence on the writing dose. The onset temperature T_{on} shifts to higher values with increasing writing power (Supplementary Fig. 7c,d), consistent with an increased effective T_g arising from a higher crosslinking density at larger writing doses. Using a 2% shrinkage-strain criterion to define T_{on} , we extract an increase from ~90 °C at 16 mW to ~120 °C at 28 mW (Supplementary Fig. 7c,d).

To minimize uncertainty in extracting T_{on}, the cooling curves were acquired using a step-hold protocol on the same temperature-controlled stage: at each temperature step, the sample was held for 5 min to reach a quasi-steady deformation before recording the shrinkage strain. With this protocol, the T_{on} values obtained from heating and cooling are highly comparable, indicating at most a small hysteresis (Supplementary Fig. 7c,d). Finally, we performed these measurements under thermal actuation (Supplementary Fig. 7) to enable a more quantitative comparison across writing conditions; localized optical heating typically follows a Gaussian intensity profile and is sensitive to beam centering, which can introduce spatially non-uniform deformation and larger uncertainty in the extracted strain and T_{on}.

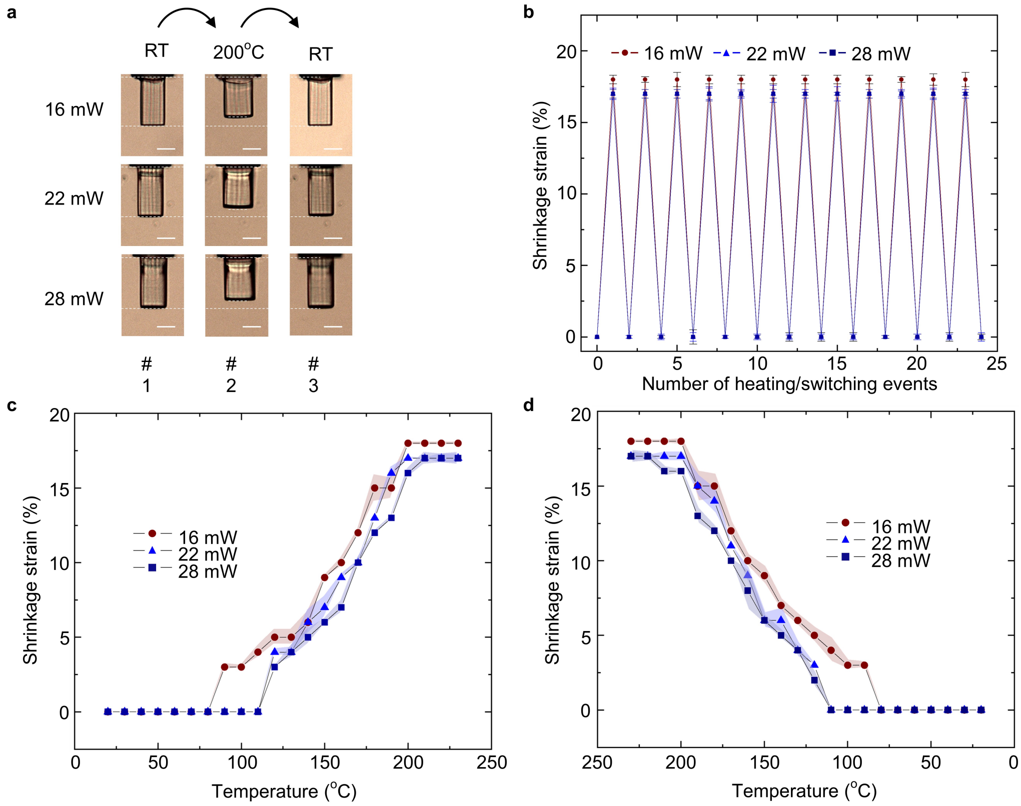

Supplementary Figure. 7 | Repeatable thermal shrinkage actuation of the suspended microstrip. a Optical micrographs of microstrip (printed at different writing powers) during thermal switching between room temperature (RT), 200 °C (on), and back to RT, **b** Peak shrinkage strain (%) recorded over repeated heating switching events (up to 24) for structures printed at 16, 22 and 28 mW. **c** Heating ramp: shrinkage strain as a function of temperature during RT→230 °C. **d** Cooling ramp: shrinkage strain as a function of temperature during 230 → RT. Shaded bands/error bars represent measurement variation across repeated trials/structures. Scale bars: 20 μm. Error bars represent the mean ± standard deviation (n = 3), obtained from repeated measurements of the same sample.

Supplementary Note 8. Mechanism of Diffusion-Guided Alignment.

Our results reveal a regime in which the final molecular alignment is decoupled from the instantaneous laser exposure. A key question is whether the observed alignment could instead arise from laser-induced alignment mechanisms (e.g., optical torque or ultrafast photochemical reorientation during exposure). We consider this unlikely based on a clear separation of timescales. With a writing speed $v = 2200\ \mu m/s$ and voxel size 0.45 μm, the effective exposure time per voxel is $t_{\text{exp}} \sim 0.2\ ms$, which is too short for substantial director reorientation of LC molecules. Moreover, higher scan speeds reduce voxel overlap, diminishing the effective area over which scan-direction shear or anchoring could be imprinted. Taken together, these considerations indicate that the LC director largely preserves its pre-exposure state during the brief writing event, rather than aligning with the laser field or scan direction. This supports a diffusion-guided picture in which the laser primarily defines the solid architecture and boundary conditions, whereas the molecular order is established after exposure through subsequent relaxation and migration processes. Diffusion-guided reorientation is well established in LC photopolymerization systems, including holographic polymer-dispersed liquid crystals (HPDLCs) and two-photon polymerization (TPP), where polymerization-induced diffusion is a central mechanism of structure formation [1]. Local monomer consumption at the focal volume generates chemical-potential (concentration) gradients that drive surrounding mesogenic/monomer components toward the exposed region. Importantly, in TPP this influx can become detrimental if not balanced by sufficient network formation: excessive or uncontrolled transport into a weakly crosslinked region may lead to swelling, distortion, or loss of geometric fidelity [2].

Consistent with this framework, our experiments indicate that reliable molecular homogenization and alignment require a sufficient relaxation and transport time window $t_{homo}^{2D} = 98\ \text{ms}$ spanning to $t_{homo}^{3D} = 3\ s$, as inferred from both 2D sheet and 3D bulk microprinting tests. To rationalize the different homogenization times observed in 2D versus 3D builds, we introduce an effective lateral transport coefficient (D_{eff}), which captures the aggregate rate at which inter-layer heterogeneity is leveled within the relevant process window. Rather than attempting to isolate an intrinsic molecular diffusion constant—expected to vary strongly with crosslink density, temperature, and local molecular order—D_{eff} provides an experimentally meaningful, process-level metric. Physically, D_{eff} encompasses (i) mass transport, where unreacted monomers/mesogens migrate along gradients generated by localized polymerization, and (ii) director relaxation, where mesogens reorient to reduce elastic free energy under anchoring constraints imposed by previously polymerized interfaces.

For example, we provide an order-of-magnitude estimate using a 1D diffusive scaling based on the mean-square displacement (MSD) relation for random-walk transport,

$$\langle x^2 \rangle = 2D_{\text{eff}}t. \tag{12}$$

Taking L as the characteristic lateral length scale that must be homogenized within a characteristic processing time window t_{homo}, we estimate

$$D_{\text{eff}} \approx \frac{L^2}{2t}. \tag{13}$$

Here, D_{eff} is introduced as an effective transport/reorientation coefficient tied to the printing geometry and the available processing window, rather than a strict intrinsic molecular diffusion constant.

Using $L = 0.2$ μm, we obtain $D_{\mathrm{eff}}^{2D} \approx 2.0 \times 10^{-13}\ \mathrm{m}^2/s$ for the 2D in-plane case with $\tau = 98\ ms$, whereas for 3D stacking a waiting time of $\tau \approx 3\ s$ is required, yielding $D_{\mathrm{eff}}^{3D} \approx 6.7 \times 10^{-15}\ m^2/s$. This decrease of D_{eff}^{3D} by about one order of magnitude is consistent with stronger suppression of transport/reorientation near repeatedly exposed, more highly crosslinked supporting features in 3D builds.

Supplementary Note 9. Triangular stacking-enabled programmed twist.

The twisting deformation is achieved by deliberately programming chirality through a triangular stacking design combined with a through-thickness flip of the director orientation. Specifically, we employ a right isosceles triangular ramp (45°-45°-90°) and initiate stacking along the 45° slanted face. The top layer is programmed with a director at +135° (relative to x-axis), such that the effective stacking/writing directions form a crossed pattern across the thickness. Upon stimulation, the two layers undergo anisotropic contraction/expansion along opposing principal axes, generating an interlayer shear mismatch and a net torsional moment; the structure accommodates this mismatch via out-of-plane rotation, resulting in a twist.

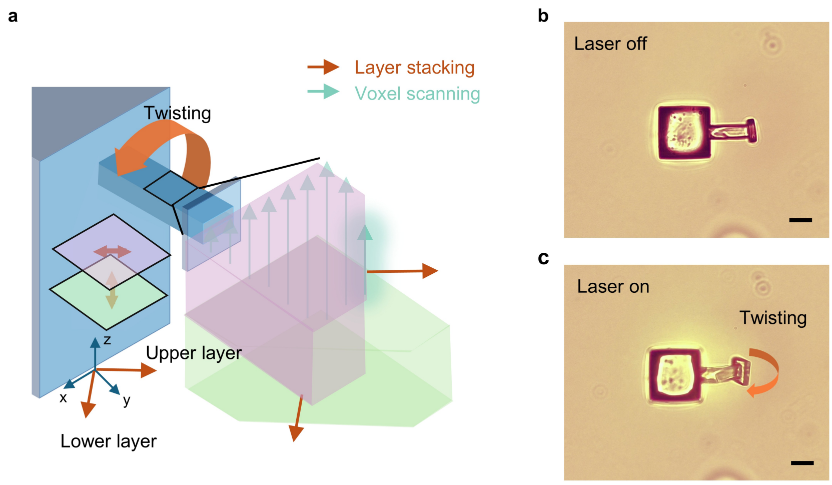

Supplementary Figure. 8 | Diffusion-guided voxel scanning and layer stacking enable programmed twisting actuation. a Schematic illustrating the writing strategy in which the voxel-scanning volume is tilted by 45° (pink), producing a block with 45° side facets (with respect to x-axis). The direction of the layer stacking is along the 135° direction, resulting in the LCN director of 135°. The bottom stacking direction (green) is programmed with an opposing −45° and the LCN director is 45°. **b** Optical micrograph of the structure in the relaxed state (laser off). **c** Optical micrograph under photothermal stimulation (laser on). Scale bar: 20 μm.

Supplementary Note 10. 3D stacking on non-planar substrates.

The layer-by-layer stacking strategy extends beyond simple rectangular builds and enables fully 3D architectures to be fabricated and anchored on non-planar substrates. This capability allows programmable LCN microstructures to be integrated onto tilted or curved topographies, forming functional interfaces reminiscent of biological cilia or sensory tentacles. As an example, a snail-inspired LCN microactuator fabricated by longitudinal stacking mimics head retraction and exhibits ~20% reversible uniaxial contraction under light stimulation (Supplementary Fig. 9a and Supplementary Movie 6). In addition, out-of-plane bending along an inclined axis is readily achieved on a tilted

surface, showing rapid, mechanically robust, and fully reversible deformation under repeated optical stimulation (Supplementary Fig. 9b and Supplementary Movie 7).

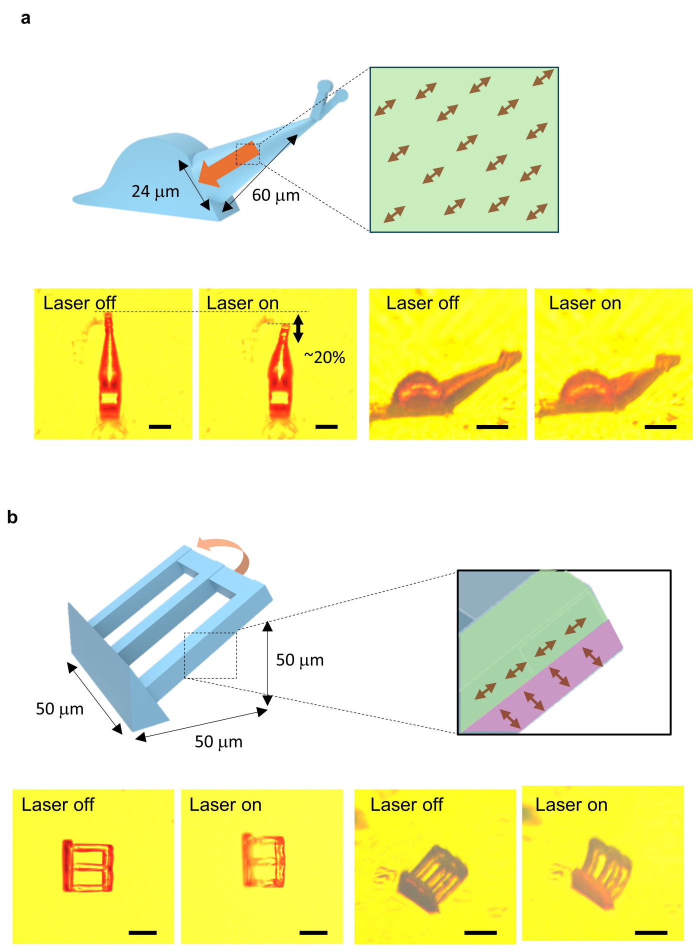

Supplementary Figure. 9 | Biomimetic light-responsive 3D/4D LCN-based microactuators. a Schematic illustration of a snail-inspired contractile LCN-based microactuator. The inset highlights the LC director fields (brown arrows) aligned along the longitudinal axis in the head region. Optical microscopy (OM) images show reversible contraction under laser irradiation. **b** Comb-like LCN-based microstructure demonstrating out-of-plane bending deformation, with dual-alignment director fields oriented at tilted angles (brown arrows). All scale bars represent 25 μm.

Supplementary Reference:

[1] Bowley, C. C., & Crawford, G. P. Diffusion kinetics of formation of holographic polymer-dispersed liquid crystal display materials. *Appl. Phys. Lett*. **76,** 2235 (2000).

[2] Zeng, H. et al. High-resolution 3D direct laser writing for liquid-crystalline elastomer microstructures. *Adv. Mater*. **26,** 2319-2322 (2014).
