# Supplementary material for: Diffusion-guided 4D microprinting of soft microactuators
Source: Nat Commun. 2026 May 14;17:6427. doi: 10.1038/s41467-026-73035-x (PMC13376197; doi:10.1038/s41467-026-73035-x)
Supplement: Supplementary file 2 — Description of Additional Supplementary Files [file 41467_2026_73035_MOESM2_ESM.pdf]

Description of Additional Supplementary Files

Supplementary Movie 1. Thermoresponsive bending of radially arranged dual-alignment micropillars exhibiting inward deformation.

The video shows LCN bilayer microstructures with dual alignment arranged in a circular configuration, with adjacent pillars rotated by 45°. Upon heating, the structures synchronously bend inward toward the center. The recording is displayed at 16× speed. The temperature is ramped from RT to 220 °C and then cooled back to RT. The deformation is recorded from the top view.

Supplementary Movie 2. Light-driven contraction of a freestanding LCN-based microstrip.

The video demonstrates the contraction behavior of a suspended, horizontally aligned LCN-based microstrip under laser illumination. A manual slider in the optical path is used to toggle the 515 nm laser (8.7 mW) on and off. The deformation is captured from a top-view perspective using a 20× objective lens. The footage includes both real-time recordings and a 0.1× slow-motion segment at the end to provide a detailed visualization of the contraction dynamics.

Supplementary Movie 3. 4D morphing of a 3D-printed biomimetic LCN flower: contraction and bending of the petals.

The video shows the contraction and bending deformation of a biomimetic flower petal structure under laser illumination. A 515 nm laser (power up to 11.8 mW) was delivered through a 20× objective to irradiate the structure, with the power gradually increased to induce the deformation. The deformation is recorded from the top view.

Supplementary Movie 4. Photothermally-driven deformation behavior of programmable multi-segment LCN-based microrobotic arms: upward bending, sideward bending, and twisting actuations.

This video demonstrates diverse laser-induced deformation behaviors, including upward bending, lateral bending, and twisting. The responses are captured from both top and side views by gradually increasing the 515 nm laser power. For the top-view observation, a 20× objective was used with power up to 7.3 mW; for the side-view observation, a 10× objective was employed with power up to 14.1 mW to characterize the mechanical dynamics.

Supplementary Movie 5. Light-driven twisting in 3D-printed biomimetic LCN microarms.

The video shows the deformation of the actuator exhibiting pure twisting under laser illumination. The deformation is recorded from the top view. A 515 nm laser (power up to 7.3 mW) was delivered through a 20× objective to irradiate the structure.

Supplementary Movie 6. Snail-inspired contractile microstructure.

The video shows the contraction behavior of a biomimetic snail structure under laser illumination. In the top view, laser ON/OFF switching is achieved by inserting or removing a beam splitter to capture rapid contraction. In the side view, the laser power is gradually increased to induce continuous deformation. A 0.1× slow-motion segment is included to visualize the contraction behavior. A 515 nm laser (power: 9.6 mW) was delivered through a 20× objective for top-view observation. The 515 nm laser (power up to 29.3 mW) was delivered through a 10× objective for side-view observation.

Supplementary Movie 7. Wing-inspired ribbed microactuator: tilted bilayer with out-of-plane bending at programmable angles.

The real-time video shows the out-of-plane bending behavior of a wing-inspired ribbed microactuator composed of a tilted bilayer under laser illumination in response to a gradual increase in laser power, with the deformation recorded from both the top view and the side view.

Irradiation conditions:

Top view-wavelength of 515 nm; power increased up to 9.6 mW; objective magnification of 20×.

Side view-wavelength of 515 nm; power increased up to 34.1 mW; objective magnification of 10×.

Supplementary Movie 8. Light-driven stag beetle-inspired LCN-based microgripper for microscale manipulation.

The video shows the bending and gripping behavior of a stag beetle-inspired microgripper under laser illumination. In the first part, the mandibles bend as the laser power is gradually increased, enabling control of the opening angle. In the second part, a grasp-and-place operation is demonstrated, where the forearms grasp a spacer with a diameter of approximately 50 μm and place it onto an LCN square-pillar platform. To enable controlled positioning, the microgripper is fabricated on the sidewall of a UV-cured resin and manually manipulated using tweezers. The deformation is recorded from the top view. A 515 nm laser (power up to 7.9 mW) was delivered through a 20× objective for the bending test, while a 515 nm laser (power up to 82 mW) was delivered through a 5× objective for the grasp-and-place operation.

Supplementary Movie 9. Target-selective activation of a smart LCN microgripper.

The video shows the target-selective actuation of the LCN microgripper under bare-fiber laser irradiation at a power of 113 mW. When a highly scattering object approaches the gap between the two LCN pillars, the enhanced scattered light activates the structure and induces inward bending for target capture. Under low scattering, no obvious bending is triggered. The deformation is recorded by camera from the side view.
