# Supplementary material for: Diffusion-guided 4D microprinting of soft microactuators
Source: Nat Commun. 2026 May 14;17:6427. doi: 10.1038/s41467-026-73035-x (PMC13376197; doi:10.1038/s41467-026-73035-x)
Supplement: Supplementary file 12 — Transparent Peer Review file [file 41467_2026_73035_MOESM12_ESM.pdf]

nature portfolio

Peer Review File

Diffusion-guided 4D microprinting of soft microactuators

Corresponding Author: Professor Yu-Chieh Cheng

This file contains all reviewer reports in order by version, followed by all author rebuttals in order by version.

Version 1:

Reviewer comments:

Reviewer #1

(Remarks to the Author)
Summary:
In this manuscript, the authors propose a 4D microprinting strategy that utilizes diffusion effects during two-photon polymerization to program anisotropy in LCN microstructures. The approach enables the fabrication of microactuators such as petals, bending strips, and microgrippers, showing the potential of diffusion-guided alignment as a novel design principle. The study is innovative, and the results are convincing, yet the demonstrated deformations remain relatively simple and the scalability to larger structures is uncertain. The reviewer considers that this manuscript requires a major revision before it can be considered for publications in this journal. A real-world application such as microfluidics, biological manipulation, or microrobotics to demonstrate its superiority is strongly recommended.

Major concerns:
1. In Fig.4, the authors characterized the mechanical performance of light-driven LCN microstrips. However, it remains unclear whether such mechanical performance is tunable, for example, in terms of contraction ratio (actuation strain), which would greatly enhance programmability. The authors are encouraged to provide a more systematic analysis of how different printing parameters influence the mechanical performance of the printed LCN structures.
2. The demonstrations in Fig.5-7 mainly show simple deformations like bending and contraction, and in essence convey the same principle – that is, combining horizontally and vertically aligned regions to achieve different deformation modes. To avoid redundancy and to better highlight the programmability of deformation, the authors are encouraged to reorganize and possibly consolidate these demonstrations into a more concise presentation.
3. Although the proposed strategy enables voxel-level printing, the fabricated LCN actuators essentially rely on stacking films with relatively simple alignment states. As a result, true voxel-level mesogen alignment programming has not yet been demonstrated. The authors are encouraged to discuss whether more sophisticated alignment modes – such as combining horizontal orientations along different in-plane directions, vertical alignment, and even regions with distinct actuation strains – can be integrated within a single continuous LCN film.
4. The demonstrations in Fig.4-7 are relatively simple and remain at the proof-of-concept level. While they effectively showcase the feasibility of the proposed strategy, they do not yet demonstrate its practical value. The authors are strongly encouraged to explore potential real-world applications of their approach – for example, in microfluidics, biological manipulation, or microrobotics.

Minor concerns:
1. The requirement of > 3 s inter-line delay for stable orthogonal alignment is mentioned, but no systematic analysis is provided. A more detailed study of inter-line delay and related parameters is encouraged.
2. The diffusion-induced alignment is not perfectly uniform within the printed structures, and its effect weakens with increasing thickness. The authors are encouraged to analyze this limitation and discuss whether the strategy is suitable for fabricating somewhat larger structures.
3. The authors are encouraged to discuss possible strategies to minimize or avoid defects like the rim-like protrusion at the mid-plane interface, and, if these protrusions influence deformation, to perform repeatability tests to evaluate their impact.

Reviewer #2

(Remarks to the Author)
To address the challenges in micro-architected 4D printed soft actuators in terms of limited architectures and deformation modes, the authors proposed a new mechanism of leveraging diffusion, rather than suppressing, for guiding the spatially

programmed volumetric alignment in 3D LCN micro-architectures. The authors also explored the 4D printing and photothermal actuation of various architectures for multi-directional deformation and actuation, including contraction, bending, and twisting, as well as demonstration in micro-manipulation.
Overall, this is very interesting work in addressing the aforementioned challenges through the new diffusion-guided mechanism for alignment in 3D LCN micro-architectures for programmable 4D printing and actuation. Achieving multimodal deformation and actuation is rather challenging at microscale. The authors have done a very nice work in addressing the challenge and showing very nice proof-of-concept demonstration. This work is novel and solid. The reviewers recommend its acceptance for publication in Nat .Commun. Minor comments are listed for further improvement.
1) For the introduction, it is recommended to add one paragraph discussing the limitations and advances in soft micro-actuators not limited to LCN based. How this work will address the limit and advance the field. The authors can refer to some work by Dr. Itai Cohen.
2) Information about response times is missing from all demonstrations. These metrics are critical for assessing the practical utility of microactuators.
3) Figures 5 and 7 present flower-like and microgripper structures. There are similar demonstrations at similar scales, such as the Photonic Microhand with Autonomous Action (Martella et al.) or Liquid Crystal Elastomer Based Thermal Microactuators and Photothermal Microgrippers Using Lateral Bending Beams (Potekhina et al.). Discussions on the comparison and advantage of this work are suggested to be included.
4) Reported actuation strains (18% contraction) are not benchmarked against existing LCE/LCN microactuators.
5) In Fig. 6d, it is unclear how the twisting deformation is achieved. Additionally, in Fig. 6, the rationale for requiring a square cell geometry is not explained.
6) Characterization data are absent, including: viscosity of the ink versus shear rate; actuation strain versus temperature.
Minor comments:
Fig. 1b, 4b, 5a, 7c, are blur. Replace with clear images.

Reviewer #3

(Remarks to the Author)
Manuscript title: Diffusion-guided 4D microprinting of soft microactuators
Authors: Wei-Ting Hsu, Po-An Tsou, Hsin-Jung Chou, Tsung-Kai Lin, and Yu-Chieh Cheng
Recommendation: Major Revision

Summary

The manuscript reports a novel diffusion-guided alignment mechanism for the fabrication of 3D liquid crystal network (LCN) microactuators via two-photon polymerization (TPP). Instead of suppressing mesogen diffusion during polymerization, the authors exploit it as a design tool to encode spatially programmed director fields, thereby enabling volumetric alignment within complex 3D microarchitectures. The work demonstrates a range of microactuators—micropillars, freestanding microstrips, flower- and microgrippers—capable of light-driven 4D transformations.
The concept is original and potentially impactful in the field of soft matter and microrobotics. However, the manuscript in its present form suffers from a number of conceptual inaccuracies, inconsistent physical descriptions, and incomplete quantitative analysis.

Strengths
• Innovative approach exploiting diffusion as a constructive design parameter in TPP.
• Technically well-executed fabrication and convincing microactuation demonstrations.
• Aesthetically appealing analogy with biological morphogenesis.
• Complete supplementary information with fabrication details and optical characterization.

Weaknesses
• Explanation of diffusion-guided alignment is largely qualitative.
• Quantitative optical analysis (birefringence/OPD) contains mathematical and conceptual errors.
• Figures and terminology (e.g., “inter-line” instead of “inter-layer” delay, diffusion direction) are sometimes ambiguous.
• Some experimental results (e.g., long-term actuation behavior) are not critically discussed.
Overall, the manuscript presents an intriguing idea but requires a major revision focusing on physical justification, quantitative rigor, and clarification of terminology.

Mandatory revisions

Physical mechanism of diffusion-guided alignment
The central claim—that mesogens reorient perpendicular to the printed surface via diffusion—remains qualitative.
A quantitative model describing diffusion length scales, reorientation times, and competition with laser-induced anchoring is required.

Incomplete and inaccurate literature contextualization

Statements such as “diffusion-driven mesogen reorientation occurs orthogonally to the laser scanning direction” and “true 3D alignment has not yet been achieved” (page 2, lines 42-44) are not accurate. Earlier works (refs 46, 47 and 48) have demonstrated “parallel” reorientation with respect to the laser scanning directions, as well as 3D twisted or helicoidal

alignment in TPP-written LCNs.
Revise the Introduction to properly acknowledge previous demonstrations of 3D or diffusion-induced alignment and clarify how the present approach advances beyond them.

Ambiguity in terminology

The text alternates between "sidewall diffusion" (for single layers) and "bulk diffusion" (for 3D structures) without defining the transition criterion.
The phrase "unreacted mesogens diffuse into the polymer" is questionable; clarify the diffusion direction and driving gradients.
Define "voxel-level scanning" precisely.
In several sentences, "inter-line delay" is used instead of "inter-layer delay".
Figures 2–3 should explicitly indicate voxel scanning direction, layer stacking, and diffusion flow with consistent color coding.

Optical characterization and birefringence analysis

In supplementary Fig. 2b, top (orange marks) and bottom (green marks) sidewalls of the square LCN microstructure are both orange on the right side (triangles). On the OPD scale, opposite OPD (-500 and + 500 nm) are associated with the bottom and top sidewalls. The authors should comment on the meaning of different signs in the OPD for the two regions which should exhibit parallel director alignment.
Equations (6)–(10) in the Supplementary Information are incorrect: the optical axis is confused with the propagation direction, and ordinary and extraordinary refractive indices are swapped.
Equation (11) cannot be derived from the preceding relations. Revise the full derivation and verify consistency with uniaxial birefringence theory.
Extracted OPD and tilt angles in Supplementary Table 1 are given with unrealistic precision despite large color bandwidths; provide error estimates.
Clarify the mapping procedure from POM colors to OPD and specify the calibration source.

Quantitative validation and missing data

Provide estimated diffusion coefficients, characteristic times, and polymerization rates.
Discuss whether diffusion-guided reorientation persists for smaller voxel sizes than measured in this work ("449 nm in width and 1495 nm in height") or reduced laser doses.
In Fig. 3d, explain the unexpected OPD "bright rim" (~1650 nm → 720 nm → 1650 nm) within the same region; the current interpretation is unclear.

Long-term actuation stability

In Fig. 4b, the actuation amplitude decreases markedly over repeated cycles.
Please discuss possible causes (e.g., photothermal fatigue, dye photobleaching, polymer relaxation) and quantify the performance degradation.

Data presentation and figure consistency

In several figures (e.g. Fig 5g) the director field is depicted as a vector, pointing in opposite direction with respect to the diffusion. Do the authors mean that mesogenic alignment is polar ?
In Fig. 3e, the bottom-left region unexpectedly brightens after development—explain whether vertical alignment is preserved or altered.

Version 2:

Reviewer comments:

Reviewer #1

(Remarks to the Author)
The authors have already addressed all my questions and concerns, I therefore, recommend the acceptance of this manuscript. Again, this is a very decent work.

Reviewer #2

(Remarks to the Author)
The reviewer's minor comments on the introduction, response time, comparison with previous studies on the soft actuators, and other characterization are well addressed in the revised version. The reviewer recommends its acceptance for publication as is.

Reviewer #3

(Remarks to the Author)
Based on an evaluation of the revised manuscript, supporting information, and the rebuttal letter, the authors have improved the technical rigor and linguistic consistency of their work. They have systematically addressed the reviewers' concerns by updating terminology, providing quantitative models, and adding new experimental validations. However, there are still technical improvements to be made before the manuscript before it can be considered for publications in this journal.

• It is recommended that authors submit their manuscripts for thorough review by a colleague who is a native speaker or proficient in academic English. This is due to the fact that some sentences appear to be convoluted and difficult to comprehend.
• “LC monomer” in Figure 1 and its caption, as well as elsewhere in the text (e.g. Line 341) whenever it is referred to the specific compound should be changed in “RM105”.
• Line 46. Please check “volumetric4D volumetric”.
• Line 92. “scanning along the x-axis 0.2 micron”. In Fig. 2 laser is scanned along the y-axis. 0.2 micron should be removed.
• Lines 126-127. In reviewer’s opinion, “y-direction” and “y” should be changed into “x-direction” and “x” as the polymerized ribbons are parallel to the x-axis.
• Lines 169-170. In the reviewer’s understanding the supporting base is a single layer. How the "inter-layer" delay should apply in this case?
• Line 180. “line” should be “layer”, as 3s is the inter-layer delay.
• Line 190. Please rephrase “….during the voxel scanning during the change…”
• Line 192. The sentence “d” is not complete: “micropillar before ……” ?
• Line 202 and beyond. “strain of about 17.4%, with 17.1% already achieved..”. The authors here refer to the change in length of a 55 micron microstrip, for which a 0.1% variation corresponds to 55nm, well beyond the optical resolution in Fig. 4a. The authors should consider the true measurement uncertainty.
• Inappropriate use of significant digits occurs also in the voxel dimensions. The authors state that the resulting voxel dimensions were measured to be “approximately 449 nm in width and 1.495 µm in height”. Reporting a voxel dimensions to the nearest nanometer is unrealistic for experimental DLW characterization. Typical laser-written voxels are subject to fluctuations in laser power, local resin heterogeneity, and polymerization shrinkage, which generally limit repeatable precision to the 10–50 nm range at best.
• Line 209. “differencing” should be “different”
• Line 230. “R” should probably be “RT”.
• Line 235. “blue region” should become “pink region”

Open Access This Peer Review File is licensed under a Creative Commons Attribution 4.0 International License, which permits use, sharing, adaptation, distribution and reproduction in any medium or format, as long as you give appropriate credit to the original author(s) and the source, provide a link to the Creative Commons license, and indicate if changes were made.
In cases where reviewers are anonymous, credit should be given to 'Anonymous Referee' and the source.
The images or other third party material in this Peer Review File are included in the article’s Creative Commons license, unless indicated otherwise in a credit line to the material. If material is not included in the article’s Creative Commons license and your intended use is not permitted by statutory regulation or exceeds the permitted use, you will need to obtain

permission directly from the copyright holder.
To view a copy of this license, visit https://creativecommons.org/licenses/by/4.0/

Reviewer #1 (Remarks to the Author):

Summary:

In this manuscript, the authors propose a 4D microprinting strategy that utilizes diffusion effects during two-photon polymerization to program anisotropy in LCN microstructures. The approach enables the fabrication of microactuators such as petals, bending strips, and micrograppers, showing the potential of diffusion-guided alignment as a novel design principle. The study is innovative, and the results are convincing, yet the demonstrated deformations remain relatively simple and the scalability to larger structures is uncertain. The reviewer considers that this manuscript requires a major revision before it can be considered for publications in this journal. A real-world application such as microfluidics, biological manipulation, or microrobotics to demonstrate its superiority is strongly recommended.

Major concerns:

R#1-1. In Fig.4, the authors characterized the mechanical performance of light-driven LCN microstrips. However, it remains unclear whether such mechanical performance is tunable, for example, in terms of contraction ratio (actuation strain), which would greatly enhance programmability. The authors are encouraged to provide a more systematic analysis of how different printing parameters influence the mechanical performance of the printed LCN structures.

Response: We agree that tunability of mechanical performance is crucial for improving the programmability of light-driven LCN microstructures. In response to the reviewer's comment, we performed additional systematic experiments to quantify how a key printing parameter—TPP laser writing power—affects the actuation behavior of dual-alignment LCN microstrips. By varying the writing power (16, 22, and 28 mW), we deliberately tuned the effective crosslinking density of the printed microstrips. We performed these measurements using thermal actuation (Supplementary Fig. 7), instead of using the optical actuation conditions shown in the previous Fig. 4, where the microstructures were optically driven. We chose thermal actuation here to enable a more quantitative and reproducible comparison across printing conditions, because local optical heating typically produces a Gaussian intensity distribution, which can lead to spatially non-uniform deformation and increased measurement uncertainty—particularly if the beam is not perfectly centered on the microstrip—thereby complicating quantitative comparisons of actuation strain and T_{on} among different writing powers.

Accordingly, we added the following description to the revised manuscript (lines 214–220):" *As shown in Supplementary Fig. S7, increasing the TPP writing power (16 – 28 mW) increases the effective crosslinking density of the printed network, raising* T_g *and shifting the onset temperature* T_{on} *of the LCN microstrips to higher values. Within this printable/processable dose window, the achieved ~17.4 % actuation strain falls within the reported strain envelope of LCN microactuators, indicating performance comparable to state-of-the-art benchmarks[51]. Further increasing contraction may be possible using longer-chain monomers, but would require careful dose calibration to balance crosslink density and* T_g *without compromising print stability.* "

R#1-2. The demonstrations in Fig.5-7 mainly show simple deformations like bending and contraction, and in essence convey the same principle – that is, combining horizontally and vertically aligned regions to achieve different deformation modes. To avoid redundancy and to better highlight the programmability of deformation, the authors are encouraged to reorganize and possibly consolidate these demonstrations into a more concise presentation.

Response: We appreciate the reviewer's suggestion. To reduce redundancy and better emphasize deformation programmability, we have reorganized Figs. 5–7 and moved several repetitive demonstration panels to the Supplementary Information. The revised main-text figures now focus on a concise set of representative modes that best highlight the distinct design rules (i.e., how horizontally and vertically aligned domains are combined and stitched to program bending, contraction, twisting, and grasping), while additional variants and supporting examples are provided in the Supplementary Figures/Videos for completeness.

R#1-3. Although the proposed strategy enables voxel-level printing, the fabricated LCN actuators essentially rely on stacking films with relatively simple alignment states. As a result, true voxel-level mesogen alignment programming has not yet been demonstrated. The authors are encouraged to discuss whether more sophisticated alignment modes – such as combining horizontal orientations along different in-plane directions, vertical alignment, and even regions with distinct actuation strains – can be integrated within a single continuous LCN film.

Response: We thank the reviewer for this suggestion. To clarify the mechanism of in-plane alignment programming, we have added a new experiment utilizing a 2 μm thin cell (Supplementary Fig. S5). By restricting the thickness, we eliminate 3D volumetric gradients and focus exclusively on the interaction between the polymerized front and the surrounding resin. Our results demonstrate that the mesogen ordering is dictated by the diffusion front originating from the sidewall of the previously polymerized segments. The resulting directors are oriented perpendicularly to the programmed boundary of each hexagon edge, independent of the surrounding lattice geometry. Local Control: This confirms that each voxel/line segment can independently prescribe its local director, enabling the integration of multi-directional alignment within a single continuous film. This level of control is verified via POM, showing distinct birefringence and color shifts. This capability allows for sophisticated optical applications, such as the local control of the retardation slow axis across a continuous LCN film.

We added the following description to the revised manuscript (Lines 182-186) and the results in Supplementary Fig. S5: "*Beyond enabling mechanically programmable actuators, the same dual-alignment, diffusion-guided stacking concept can also provide tunable spatial patterning of in-plane director fields in continuous LCN films, with potential relevance to optical functionalities such as local retardation-axis control. As a proof of concept, we patterned a honeycomb lattice in a 2 μm-thick cell to demonstrate localized control of in-plane alignment in a micrometer-thick LCN film (Supplementary Fig. S5).*"

R#1-4 The demonstrations in Fig.4-7 are relatively simple and remain at the proof-of-concept level. While they

effectively showcase the feasibility of the proposed strategy, they do not yet demonstrate its practical value. The authors are strongly encouraged to explore potential real-world applications of their approach – for example, in microfluidics, biological manipulation, or microrobotics.

Response: We appreciate the reviewer's suggestion and agree that the demonstrations in Figs. 4–7 are close to the proof-of-concept stage. However, we would like to emphasize that our platform is already compatible with an application-oriented scenario in biological micromanipulation, as we demonstrate a light-driven microgripper that can capture a micro-object (as a model cargo) and place it at a designated location, representing a practical pick-and-place workflow enabled by programmable actuation and wireless optical control. Furthermore, we agree that microfluidic integration is an important next step; although we have not yet implemented a fully enclosed microfluidic chip due to facility limitations, we have explored a practical, biology-oriented experimental configuration by mounting/operating our printed LCN structures on a concavity (depression) glass slide that is widely used for biological observation.

In the revised manuscript, we added a new experiment with the following description (lines 289-198): "*We further demonstrate a scalable, application-oriented self-activated LCN microgripper integrated on a cell-culture cavity slide, providing a working clearance (cell gap) of ~700 μm. Using DLW with an air objective, we print a larger-voxel architecture with dz = 10 μm and a 0.5 μm hatch spacing (72 mW, 1200 μm/s), yielding a gripper of ~160 μm × 60 μm × 600 μm. The device exhibits target-triggered autonomous grasping: a bottom-up fiber laser (515 nm, 113 mW) is aligned through the gap between the two pillars to avoid direct irradiation, and closure occurs only when a highly scattering (high-reflectance) target enters the gap, where the high scattered target enhances local photothermal deposition via scattering-mediated coupling. In contrast, transparent or weakly scattering targets do not trigger actuation. Turning the laser off reopens the grippers and releases the target, enabling a directly translatable route to event-driven micromanipulation for microfluidics and cell handling.*"

Minor concerns:

R#1-1.1 The requirement of > 3 s inter-line delay for stable orthogonal alignment is mentioned, but no systematic analysis is provided. A more detailed study of inter-line delay and related parameters is encouraged.

Response: We thank the reviewer for this suggestion and acknowledge that our earlier draft used the term "inter-line delay" imprecisely. In the revised manuscript, we clarify that the reported criterion $\Delta t \gtrsim 3$ refers to the layer-to-layer (inter-layer) time window—i.e., the minimum interval after completing one layer's line-by-line scanning before stacking/writing the subsequent layer—during which diffusion-driven homogenization and interfacial reorientation can equilibrate.

The description is added in the revised manuscript (lines 154-156): " *Accordingly, the layer-by-layer stacking time—set by the overall design and structure size—should exceed the characteristic diffusion time, so that diffusion-driven reorientation can equilibrate before the next layer is written, ensuring consistent diffusion-guided alignment upon subsequent stacking.*"

To further rationalize this delay requirement, we additionally provide a quantitative, process-relevant

analysis in Supplementary Note 8, where we introduce an effective lateral transport/reorientation coefficient. This analysis explicitly links the required inter-line delay to the characteristic homogenization length scale L (set by the hatch spacing) and to the available processing window τ, and explains why the necessary delay increases substantially in 3D overlapped builds: repeated exposure and overlap increase the local crosslinking density near supporting features, which suppresses transport and director relaxation and therefore requires a longer time to reach quasi-uniform alignment before the next line/layer is written.

R#1-1.2 The diffusion-induced alignment is not perfectly uniform within the printed structures, and its effect weakens with increasing thickness. The authors are encouraged to analyze this limitation and discuss whether the strategy is suitable for fabricating somewhat larger structures.

Response: We thank the reviewer for this important point. We agree that diffusion-guided alignment may not be perfectly uniform in thicker builds, which can be sensitively reflected in the POM colour/contrast. Achieving fully uniform optical performance would require further optimization of writing conditions, for example, depth-dependent dose tuning (deeper writing may require slightly higher power due to attenuation/absorption in the precursor and partial shadowing by previously polymerized features). The apparent non-uniformity can also be amplified by post-development shrinkage and minor shape distortions (e.g., local tapering/tilt), which modulate local thickness/OPD even when the underlying director field is only slightly perturbed. In addition, improving geometric stability during handling/observation—e.g., by keeping the cover glass in place after development—may help preserve the intended architecture and maintain more consistent optical conditions. Accordingly, for applications demanding high optical precision across thick volumes, a dedicated study of depth-dependent dose compensation and systematic depth mapping will be required.

Nevertheless, the primary purpose of this work is to demonstrate designable, programmable actuation that remains functional even with small alignment deviations. Experimentally, we retain effective alignment programming and robust bending actuation up to ~60 μm in height using an oil-immersion objective, and further demonstrate bendable pillars as tall as ~600 μm without depth-dependent dose compensation, using an air objective with a larger working distance (new experiment, Fig. 6b).

R#1-1.3 The authors are encouraged to discuss possible strategies to minimize or avoid defects like the rim-like protrusion at the mid-plane interface, and, if these protrusions influence deformation, to perform repeatability tests to evaluate their impact.

Response: We thank the reviewer for highlighting the rim-like protrusion near the mid-plane interface. In our designs, this feature is largely intentional: to improve mechanical stability and ensure reliable domain stitching, we deliberately repeat the inter-layer region (typically 1–3 hatch-spaced interlayers) before switching to the orthogonal alignment domain. This reinforcement was motivated by a practical fabrication concern—when writing a suspended structure and then turning to write the sidewalls of an orthogonally programmed layer, the unsupported free end can slightly drift, leading to misregistration and imperfect bonding at the junction. Empirically, introducing several repeated interlayers reduces this positional deviation and enables robust connection; the optimal number of such interlayers depends on the specific geometry and the degree of

suspension in the target structure.

The added description (lines 244-247) is "... *To further enhance mechanical stability and ensure reliable stitching when transitioning between orthogonal alignment domains, we intentionally insert a short-reinforced junction by repeating the inter-layer region (typically 3-5 hatch-spaced interlayers) before switching the stacking direction during fabrication of the suspended petals.*"

Reviewer #2 (Remarks to the Author):

To address the challenges in micro-architected 4D printed soft actuators in terms of limited architectures and deformation modes, the authors proposed a new mechanism of leveraging diffusion, rather than suppressing, for guiding the spatially programmed volumetric alignment in 3D LCN micro-architectures. The authors also explored the 4D printing and photothermal actuation of various architectures for multi-directional deformation and actuation, including contraction, bending, and twisting, as well as demonstration in micro-manipulation. Overall, this is very interesting work in addressing the aforementioned challenges through the new diffusion-guided mechanism for alignment in 3D LCN micro-architectures for programmable 4D printing and actuation. Achieving multimodal deformation and actuation is rather challenging at microscale. The authors have done a very nice work in addressing the challenge and showing very nice proof-of-concept demonstration. This work is novel and solid. The reviewers recommend its acceptance for publication in Nat .Commun.
Minor comments are listed for further improvement.

R#2-1 For the introduction, it is recommended to add one paragraph discussing the limitations and advances in soft micro-actuators not limited to LCN based. How this work will address the limit and advance the field. The authors can refer to some work by Dr. Itai Cohen.

Response: We sincerely thank the reviewer for this insightful suggestion. We agree that contextualizing our work within the broader landscape of soft micro-robotics—beyond just LCN-based systems—greatly strengthens the impact of our manuscript. As suggested, we have added a new discussion in the Introduction (Lines 48–68 in the modified manuscript) that highlights a universal challenge in the field: the fundamental trade-off between actuation complexity (degrees-of-freedom) and structural footprint (scaling). We specifically contrasted our approach with the state-of-the-art in micro-origami and electronically integrated micro-robots, including the pioneering work of Dr. Itai Cohen [50, 51]. While these platforms achieve digital precision, they often rely on surface-dominated architectures or discrete mechanical components (hinges/circuitry) that consume significant functional volume, limiting their deployment in confined spaces. Our work addresses this limitation by introducing 3D volumetric programming. By embedding actuation logic directly into the molecular orientation field through a diffusion-guided strategy, we eliminate the need for bulky mechanical overhead, high-cost fabrication, or post-manipulations. This advances the field by enabling monolithic, high-density actuators capable of complex, multi-axis morphing.

The description is added in the introduction of the revised manuscript (lines 48-68): " *Beyond LCN-based microstructures, all soft microrobots suffer from a fundamental trade-off between geometric complexity and actuation density. Specifically, generating fundamental motions such as bending or twisting necessitates a precisely programmed orthogonal orientation field within the material's cross-section — a requirement that is notoriously difficult to satisfy at micro-scales. While pioneering work in micro-origami and thin-film actuators can offer digital precision through sophisticated semiconductor fabrication[50-51], these platforms are often constrained by their surface-dominated architectures. Consequently, achieving complex, multi-axis activation typically requires dense arrays of discrete hinges and control circuitry, which increases the mechanical overhead and*

limits deployment within confined geometries. To achieve high functional density within a soft, monolithic body, 3D volumetric programming, which embeds actuation logic directly into the molecular orientation field, represents a powerful paradigm shift. Nonetheless, realizing such a capability remains a formidable challenge, as current fabrication platforms struggle to encode the required orthogonal gradients in situ without cumbersome post-manipulations.

Here, we report a diffusion-guided 3D volumetric printing strategy that overcomes these scaling and complexity limitations by embedding high-density actuation logic directly into the evolving molecular field. Specifically, we harness LC monomer diffusion through layered TPP architectures to encode spatially programmed alignment, thereby achieving true 4D microprinting of soft microactuators. We demonstrate that molecular orientation continuously realigns perpendicular to the advancing printed surface profile; notably, at high scanning speeds, the transient laser-induced anchoring forces become insufficient to disrupt this diffusion-driven orientation process. Our proposed mechanism enables progressive, volumetric control of the LC director field, mimicking the seamless integration of form and function observed in biological morphogenesis, achieving for a monolithic, intelligent microactuators to execute coordinated, high-degree-of-freedom motions with embedded actuation logic."

R#2-2 Information about response times is missing from all demonstrations. These metrics are critical for assessing the practical utility of microactuators.

Response: We thank the reviewer for highlighting the importance of response time. We have now quantified the actuation dynamics by measuring the time-dependent contraction of the LCN microactuators under optical stimulation at multiple irradiation powers. Specifically, we tracked the normalized contraction strain as a function of time for each power level and extracted characteristic response metrics (e.g., the rise time to reach 50% and 90% of the steady-state contraction, as well as the saturation contraction level). As shown in the added plot, increasing optical power accelerates the response and increases the attainable contraction until it approaches a plateau at higher powers, providing a clear power–speed–strain trade-off that is critical for practical operation. These response-time data and the corresponding analysis have been added to Supplementary Fig. 6a and described in the revised manuscript to enable a more complete assessment of the actuator utility across operating conditions.

The new measurement is added in the revised manuscript (lines 202-206): " *To enable photothermal actuation (see Methods), we introduced an azo-dye-based additive after development. The contraction of the suspended microstrip (Supplementary Video 2) demonstrated a rapid response time under photothermal actuation, reaching an apparent steady-state strain within 50 ms (approximately 3 frames at 60 fps) as show in Supplementary Fig. S6a.*"

R#2-3 Figures 5 and 7 present flower-like and microgripper structures. There are similar demonstrations at similar scales, such as the Photonic Microhand with Autonomous Action (Martella et al.) or Liquid Crystal Elastomer Based Thermal Microactuators and Photothermal Microgrippers Using Lateral Bending Beams (Potekhina et al.). Discussions on the comparison and advantage of this work are suggested to be included.

Response: We thank the reviewer for pointing out prior microgripper demonstrations at similar length scales. While these works compellingly demonstrate microscale grasping using light- or heat-driven soft materials, our contribution focuses on a different bottleneck: how to reliably program spatially defined

director/alignment states during 3D microfabrication without relying on predefined molds/microgrooves, post-fabrication assembly, or a single globally uniform alignment across the entire structure**.** Therefore, we add a paragraph in the manuscript to address the advantage of our proposed work compared to these two works.

We added the discussion and these two literatures in the revised manuscript (lines 286-289): " *Distinct from conventional LCN microgrippers and microhands that necessitate intricate pre-alignment layers, micro-cell replica molding*[52]*, or laborious post-assembly micromanipulation*[53]*, our platform enables one-step, in situ fabrication with functionality programmed during printing, eliminating manual post-processing.*"

R#2-4 Reported actuation strains (18% contraction) are not benchmarked against existing LCE/LCN microactuators.

Response: We thank the reviewer for this important suggestion. The previously reported ~18% actuation strain was obtained under optical actuation. However, we found that repeated optical irradiation could induce local damage due to the high peak intensity associated with the Gaussian laser profile. Therefore, we used thermal actuation for a more controlled and uniform evaluation, under which the measured actuation strain was ~17.4 %. Within this printable/processable dose window, the achieved ~17% actuation strain falls within the reported strain envelope of LCN microactuators, indicating performance comparable to representative state-of-the-art benchmarks[51]. Further increases in contraction may be possible using longer-chain monomers, but this would require careful dose calibration to balance crosslinking density and glass transition temperature (T_g) without compromising print stability.

The description is added in the revised manuscript (lines 216-218): " *Within this printable/processable dose window, the achieved ~17.4 % actuation strain falls within the reported strain envelope of LCN microactuators, indicating performance comparable to state-of-the-art benchmarks*[51]."

R#2-5 In Fig. 6d, it is unclear how the twisting deformation is achieved. Additionally, in Fig. 6, the rationale for requiring a square cell geometry is not explained.

Response: Thanks for the helpful comments. We have clarified this design principle and note that the square cell in Fig. 6 was primarily chosen as a convenient, highly symmetric tiling unit that provides consistent boundary conditions for array assembly. We additionally include a single-unit demonstration showing a clear twist response, together with a clearer illustration and description of the twisting deformation (Supplementary Fig. 8).

We added the explanation in the revised manuscript (lines 266-270): " *The square-cell junction acts as a compliant transition between neighboring segments, reducing interfacial strain mismatch and improving load transfer and mechanical robustness. More complex behaviors, such as twisting, are achieved by introducing a slanted stacking interface that programs crossed in-plane alignments via ±45° stacking of the upper and lower layers (Supplementary Fig. 8 and Supplementary Video 5).* "

R#2-6 Characterization data are absent, including: viscosity of the ink versus shear rate; actuation strain versus temperature.

Response: We thank the reviewer for these suggestions. First, viscosity versus shear rate: we agree that rheological characterization would be informative; however, we currently do not have access to a suitable rheometer and therefore cannot provide viscosity–shear-rate curves in this revision.

We have performed additional measurements and now provide the requested strain–temperature characterization in the revised Supplementary Information (Supplementary Fig. 6c,d). These data show how the shrinkage strain evolves during heating and cooling and enable extraction of the onset temperature T_{on}.

We added the following description in the main text (revised manuscript, lines 213–216): " *TPP writing dose provides a single, practical knob that jointly governs activation characteristics, mechanical integrity, and diffusion-guided alignment. As shown in Supplementary Fig. S7, increasing the TPP writing power (16–28 mW) increases the effective crosslinking density of the printed network, raising T_g and shifting the onset temperature T_on of the LCN microstrips to higher values.* "

Minor comments:

R#2-6.1 Fig. 1b, 4b, 5a, 7c, are blur. Replace with clear images.

Response: We thank the reviewer for this helpful comment. We have replaced these panels with higher-resolution, clearer images in the revised manuscript.

Reviewer #3 (Remarks to the Author):

Manuscript title: Diffusion-guided 4D microprinting of soft microactuators

Authors: Wei-Ting Hsu, Po-An Tsou, Hsin-Jung Chou, Tsung-Kai Lin, and Yu-Chieh Cheng

Recommendation: Major Revision

Summary

The manuscript reports a novel diffusion-guided alignment mechanism for the fabrication of 3D liquid crystal network (LCN) microactuators via two-photon polymerization (TPP). Instead of suppressing mesogen diffusion during polymerization, the authors exploit it as a design tool to encode spatially programmed director fields, thereby enabling volumetric alignment within complex 3D microarchitectures. The work demonstrates a range of microactuators—micropillars, freestanding microstrips, flower- and microgrippers—capable of light-driven 4D transformations.

The concept is original and potentially impactful in the field of soft matter and microrobotics. However, the manuscript in its present form suffers from a number of conceptual inaccuracies, inconsistent physical descriptions, and incomplete quantitative analysis.

Strengths

• Innovative approach exploiting diffusion as a constructive design parameter in TPP.

• Technically well-executed fabrication and convincing microactuation demonstrations.

• Aesthetically appealing analogy with biological morphogenesis.

• Complete supplementary information with fabrication details and optical characterization.

Weaknesses

• Explanation of diffusion-guided alignment is largely qualitative.

• Quantitative optical analysis (birefringence/OPD) contains mathematical and conceptual errors.

• Figures and terminology (e.g., “inter-line” instead of “inter-layer” delay, diffusion direction) are sometimes ambiguous.

• Some experimental results (e.g., long-term actuation behavior) are not critically discussed.

Overall, the manuscript presents an intriguing idea but requires a major revision focusing on physical justification, quantitative rigor, and clarification of terminology.

Mandatory revisions

R#3-1.Physical mechanism of diffusion-guided alignment

The central claim—that mesogens reorient perpendicular to the printed surface via diffusion—remains qualitative. A quantitative model describing diffusion length scales, reorientation times, and competition with laser-induced anchoring is required.

Response: We thank the reviewer for pointing out the need for a more quantitative discussion regarding the physical mechanism. We have addressed the reviewer’s concerns by analyzing the competition between laser-induced effects and diffusion-guided alignment through the lens of disparate spatial and temporal scales. Furthermore, we have introduced a quantitative model using an effective lateral transport coefficient (D_{eff}) to describe the diffusion length scales and homogenization times.

1. Separation of timescales and suppression of laser-induced effects: At our writing conditions (P = 22 mW, v = 2200 μm/s, voxel size ≈ 449 nm), the effective exposure time per voxel is t ≈ 0.2 ms. This is significantly shorter than the typical director reorientation time for LC molecules. Consequently, the LC director largely preserves its pre-exposure state during the brief writing event. Furthermore, our high scanning speed reduces the laser overlap area, diminishing any scan-direction shear or optical torque that could "imprint" a specific anchoring. This explains why the final molecular alignment is decoupled from the instantaneous laser exposure.

We add the description (lines 282-292) in Supplementary Note 8: " *Our results reveal a regime in which the final molecular alignment is decoupled from the instantaneous laser exposure. A key question is whether the observed alignment could instead arise from laser-induced alignment mechanisms (e.g., optical torque or photochemical reorientation during exposure). We consider this unlikely based on a clear separation of timescales. With a writing speed v = 2200 μm/s and voxel size 0.449 μm, the effective exposure time per voxel is t_{exp}~ 0.2 ms, which is too short for substantial director reorientation of LC molecules. Moreover, higher scan speeds reduce voxel overlap, diminishing the effective area over which scan-direction shear or anchoring could be imprinted. Taken together, these considerations indicate that the LC director largely preserves its pre-exposure state during the brief writing event, rather than aligning with the laser field or scan direction. This supports a diffusion-guided picture in which the laser primarily defines the solid architecture and boundary conditions, whereas the molecular order is established after exposure through subsequent relaxation and migration processes.*"

We also added the statement in the revised manuscript (Lines 103-106) to refer to the Supplementary Note 8: " *Within this same timescale framework, the scan-direction shear from laser-induced anchoring is unlikely to compete with diffusion-guided alignment, because diffusion/reorientation proceeds over micron-scale distances on milliseconds to seconds timescales, whereas local laser exposure and polymerization occur on sub-millisecond timescales (see Supplementary Note 8).*"

2. Diffusion length scale: While the theoretical diffusion length scale could extend to several microns, our system primarily requires effective homogenization over the sub-micron scale, specifically corresponding to the hatch spacing or inter-layer spacing (L ≈ 0.2 μm). Given this short characteristic length, the diffusion-guided mechanism is highly efficient in establishing a uniform director field within the available time window.

3. Quantitative model of diffusion and homogenization with effective lateral transport coefficient: To rationalize the observed alignment, we introduce an effective lateral transport coefficient (D_{eff}) in Supplementary Note 8. This coefficient encompasses both mass transport (monomer diffusion) and director relaxation (mesogen reorientation). we estimate D_{eff} using the diffusive scaling relation $\mathrm{D}_{eff} \approx \frac{L^2}{2\tau}$, where L $(0.2\ \mu m)$is the characteristic lateral length scale and τ is the processing time window (t_{homo}). Here, we obtain $D_{eff}^{2D} \approx 2.0 \times 10^{-13}\ m^2/s$ for the 2D in-plane case with $\tau\ =\ 98\,ms$ and a waiting time of $\tau \approx 3\ s,\ D_{eff}^{3D} \approx\ 6.7 \times 10^{-15}\ m^2/s$ for 3D stacking . This decrease of D_{eff}^{3D} by about one order of magnitude is consistent with stronger suppression of transport/reorientation near repeatedly exposed, more highly crosslinked supporting features in 3D builds. This model provides a quantitative basis for the temporal requirements observed in our experiments.

R#3-2 Incomplete and inaccurate literature contextualization

Statements such as "diffusion-driven mesogen reorientation occurs orthogonally to the laser scanning direction" and "true 3D alignment has not yet been achieved" (page 2, lines 42-44) are not accurate. Earlier works (refs 46, 47 and 48) have demonstrated "parallel" reorientation with respect to the laser scanning directions, as well as 3D twisted or helicoidal alignment in TPP-written LCNs.

Revise the Introduction to properly acknowledge previous demonstrations of 3D or diffusion-induced alignment and clarify how the present approach advances beyond them.

Response: We appreciate the reviewer's correction regarding the historical development of 3D alignment in TPP-written LCNs. We have revised the Introduction to accurately reflect the contributions of earlier works [46-48] and [49]. Specifically, we have clarified that while laser scanning trajectories can dictate molecular alignment—ranging from parallel reorientation [46, 47] to 3D twisted or helicoidal structures [48]—these methods typically rely on trajectory-dependent anchoring. As noted by the reviewer, while in situ control like DiTuM [49] enables spatially continuous gradients, it often operates through a competitive equilibrium between laser-induced axes and initial alignment conditions. Our work advances beyond these techniques by introducing a diffusion-guided mechanism that is fundamentally decoupled from the scanning trajectory at high speeds. Unlike previous methods where the alignment is "slaved" to the laser path, our approach harnesses the advancing surface profile of the layered architecture to drive molecular orientation. This allows us to encode abrupt, orthogonal, and arbitrary 3D volumetric tensors that were previously difficult to achieve without interference from the scanning direction. We have updated the manuscript (Page 2, Lines 40-46 in the modified manuscript) to clarify this distinction and better define our contribution to "true 4D volumetric microprinting.

We updated the introduction in the revised manuscript (lines 41-47): " *Recent breakthroughs demonstrated that laser scanning trajectories can dictate molecular alignment in LCN thin film*[46-47]*, a capability later extended to 3D microstructures via TPP*[48]*. However, such control remains restricted to isolated segments or planar geometries. While Directional Tuning Mode (DiTuM)*[49] *attempted spatially continuous volumetric control, its reliance on a competitive equilibrium between laser-induced axes and initial alignment results in continuous gradients rather than abrupt orthogonal orientations. This dependency on scanning trajectories precludes precise volumetric tensor control, leaving volumetric 4D volumetric microprinting with programmable arbitrary 3D director fields a persistent challenge.*"

3. Ambiguity in terminology

R#3-3.1The text alternates between "sidewall diffusion" (for single layers) and "bulk diffusion" (for 3D structures) without defining the transition criterion.

Response: We thank the reviewer for this helpful comment and for prompting clearer wording. To avoid possible misunderstanding, we clarify that the transition from "sidewall" to "bulk" diffusion is not a change in the underlying physics, but rather a shift in the dominant templating interface. We added the description of the transition criterion in the modified manuscript (lines 144-147 and 151-156): " *Once fabrication proceeds by upward layer stacking to form 3D microstructures, the dominant templating interface shifts from the sidewall of an isolated voxel-scanned line segment to the outer polymer/precursor interface of the already-built multilayer architecture. Accordingly, mobile LC monomers near this evolving interface are reoriented by interfacial templating during diffusion-guided equilibration. .... The transition criterion is not a mechanistic switch: in both regimes, alignment is templated at the polymer/precursor interface, with the effective templating surface evolving from the boundary of an isolated line segment to the outer surface of a multilayer, overlapped 3D architecture. Accordingly, the layer-by-layer stacking time—set by the overall design and structure size—should exceed the characteristic diffusion time, so that diffusion-driven reorientation can equilibrate before the next layer is written, ensuring consistent diffusion-guided alignment upon subsequent stacking.* "

R#3-3.2The phrase "unreacted mesogens diffuse into the polymer" is questionable; clarify the diffusion direction and driving gradients.

We agree that the wording "unreacted mesogens diffuse into the polymer" was imprecise and potentially misleading. We have therefore replaced "unreacted mesogens" with "LC mesogens" throughout the revised manuscript where appropriate. Our intention was to describe the redistribution of mobile LC monomers or LC mesogens within the unpolymerized precursor adjacent to the newly polymerized network.

We have revised the text to explicitly clarify the diffusion direction and the associated driving gradients (Lines 75–80), as follows: ".... *This process locally consumes reactive monomers, thereby establishing a sharp spatial gradient in chemical potential at the polymer/precursor interface. The surrounding mobile LC monomers redistribute toward the polymer/precursor interface and partially into the newly formed network region, driven by local chemical-potential gradients arising from local differences in monomer density (Fig. 1b, ii). During this*

transport process, LC mesogens near the interface realign to minimize interfacial energy, following the surface contour to form a spatially graded orientation (Fig. 1b, iii)."

R#3-3.3 Define "voxel-level scanning" precisely.

We thank the reviewer for this suggestion. In this work, "voxel-level scanning" refers to the prescribed scanning path of the DLW focal volume (i.e., the laser voxel) during fabrication. As the focal volume is translated, the printed line is formed by a sequence of partially overlapping polymerized voxels along that path. We have revised the wording in the manuscript to clarify this definition and avoid ambiguity. We have added the definition in the revised manuscript (lines 257-259): " *Fig. 4g summarizes the directions of voxel scanning, diffusion, and layer stacking during DLW fabrication of suspended LCN petals. Here, the voxel scanning refers to the prescribed scanning path of the DLW focal volume (i.e., the laser voxel) during fabrication*."

R#3-3.4 In several sentences, "inter-line delay" is used instead of "inter-layer delay".

Response: We thank the reviewer for their careful reading and for catching this terminology error. We have corrected this in the revised manuscript (Lines 174-175 and Line 178) as follows:

Lines 174-175:"..... *state in a 3D bulk build, an inter-layer delay of >3 s is required (Supplementary Fig. S3)*."

Line 178:"... *Nevertheless, this inter-layer delay of more than 3 s does not substantially increase...*"

R#3-3.5 Figures 2–3 should explicitly indicate voxel scanning direction, layer stacking, and diffusion flow with consistent color coding.

Response: We agree that explicit visual indicators significantly improve the clarity of the underlying physical processes. Following the reviewer's suggestion, we have updated Figures 2 and 3 to include standardized arrows and labels. A consistent color-coding scheme has been implemented throughout the manuscript to distinguish between the different vectors:

- ✓ Red arrows: Voxel scanning direction
- ✓ Orange arrows: Layer stacking direction
- ✓ Green arrows: Diffusion flow
- ✓ Brown indicators: Director field (molecular orientation)

The captions for Figures 2 and 3 have been updated to reflect these additions, ensuring that the spatial relationship between the scanning path, the resulting concentration gradients (diffusion), and the final molecular alignment is immediately apparent to the reader.

4. Optical characterization and birefringence analysis

R#3-4.1 In supplementary Fig. 2b, top (orange marks) and bottom (green marks) sidewalls of the square LCN microstructure are both orange on the right side (triangles). On the OPD scale, opposite OPD (-500 and + 500 nm) are associated with the bottom and top sidewalls. The authors should comment on the meaning of different signs in the OPD for the two regions which should exhibit parallel director alignment.

We sincerely thank the reviewer for pointing out the inconsistencies in the markers of Supplementary Fig. 2b. The markers were indeed misplaced in the original version, and we have now corrected them to accurately represent the sampled regions. Specifically:

- ✓ Orange markers are now placed in the center of the microstructure.
- ✓ Yellow markers represent the right boundary (vertical sidewall).
- ✓ Green markers represent the bottom boundary (horizontal sidewall).

We have selected these three positions to represent the overall alignment due to the structural symmetry (where top/bottom and left/right sidewalls exhibit identical behaviors).

R#3-4.2 Equations (6)–(10) in the Supplementary Information are incorrect: the optical axis is confused with the propagation direction, and ordinary and extraordinary refractive indices are swapped. Equation (11) cannot be derived from the preceding relations. Revise the full derivation and verify consistency with uniaxial birefringence theory.

We have thoroughly revised the full derivation in the Supplementary Information to align with standard uniaxial birefringence theory. The key corrections include:

- ✓ Refinement of Geometric Definitions: We have clearly redefined the relationship between the propagation direction (along the z-axis), the optical axis (director field), and the tilt angle (θ). The previous confusion between sin and cos terms stemmed from an inconsistent definition of the tilt relative to the substrate normal (z-axis) versus the substrate plane. This has been unified throughout the derivation.
- ✓ Refractive Index Consistency: The ordinary (n_o) and extraordinary (n_e) refractive indices have been correctly assigned according to the uniaxial LC model, ensuring that the effective refractive index n_{eff} correctly follows the index ellipsoid equation.
- ✓ Correction of Equation (11): With the corrected preceding relations, Equation (11) has been re-derived to ensure mathematical consistency.
- ✓ Update to Supplementary Table 1: We have updated the calculated tilt angles relative to the surface normal (z-axis) to ensure full consistency between our experimental OPD measurements and the revised theoretical model.

The revised Supplementary Information now provides a rigorous and consistent derivation of the optical path difference (OPD) as a function of the 3D director orientation.

R#3-4.3 Extracted OPD and tilt angles in Supplementary Table 1 are given with unrealistic precision despite large color bandwidths; provide error estimates.

Response: We agree with the reviewer that the precision of the extracted OPD and tilt angles should reflect the experimental uncertainty inherent in POM color analysis. To address this, we have revised Supplementary Table 1 to include error estimates for all calculated values. By propagating these OPD ranges through our revised equations, we have converted the color variations into corresponding tilt angle intervals.

R#3-4.4 Clarify the mapping procedure from POM colors to OPD and specify the calibration source.

Response: We thank the reviewer for requesting clarification. OPD was not determined by static visual inspection alone; instead, we used an optical compensation procedure (Fig. 2) to assign the retardation regime and then calibrated the color-to-OPD mapping. Specifically, we inserted a full-wave plate (λ = 550 nm) and performed a slow-axis switching test by alternating the orientation of the plate's slow axis relative to the sample's director. The resulting additive (higher-order) versus subtractive (lower-order) interference-color shifts provide a dynamic phase check, which resolves ambiguities in low-retardation regions (e.g., first-order gray) and in cases where similar colors can occur in different interference orders (e.g., second- and third-order blue). The observed colors were then cross-referenced with a digital Michel–Lévy chart calibrated to our microscope illumination and white-balance settings; the bandwidth of the additive/subtractive shifts was used to estimate OPD uncertainty and propagate error bars for the extracted tilt angles (Supplementary Table 1).

We add the description in the supplementary (lines 148-154): " *To ensure the accuracy of the extracted optical path difference (OPD) and the derived director tilt angles, the interference colors were validated using the optical compensation method. By alternating the orientation of a full-wave plate's (λ = 550 nm) slow axis relative to the microstructure, we observed the resulting additive and subtractive color shifts. This procedure allowed us to unambiguously determine the specific retardation regime and provide realistic error estimates for the molecular orientation. Consequently, the reported OPD values are not merely based on static visual mapping but are cross-verified through dynamic phase-shift identification, ensuring the reliability of the 3D alignment characterization*."

5. Quantitative validation and missing data

R#3-5.1 Provide estimated diffusion coefficients, characteristic times, and polymerization rates.

Response: We thank the reviewer for this request. We note, however, that the key transport phenomenon in our work occurs during 3D stacking in an already partially polymerized, repeatedly exposed architecture, where local crosslinking gradients and interfacial anchoring can strongly hinder migration and reorientation. Under these conditions, reporting a single "intrinsic" molecular diffusion coefficient (as typically discussed for an isolated written line in prior literature) is not fully representative. Instead, we quantify a process-relevant effective lateral transport/reorientation coefficient, D_{eff}, which captures how rapidly inter-line/inter-layer heterogeneity is homogenized within the available processing time window and thus directly relates to the printing outcome.

Using a 1D diffusive scaling $\mathrm{D}_{eff} \approx \frac{\mathrm{L}^2}{2\mathrm{t}}$ with the hatch-defined length scale L = 0.2 μm, we estimate $D_{eff}^{2D} \approx$ $2.0 \times 10^{-13}\ m^2/\mathrm{s}$ for the 2D case (t = 98), whereas 3D overlapped stacking requires a longer equilibration window ($\mathrm{t} \approx$ 3 s), yielding $D_{eff}^{3D} \approx\ 6.7 \times 10^{-15}\ m^2/\mathrm{s}$, consistent with stronger suppression of transport/reorientation near repeatedly exposed supporting features. In addition, the polymerization/exposure timescale is much faster than transport: at v = 2200 μm/s and voxel length ~ 0.449 μm, the effective exposure time is $t_{exp} \approx$ 0.20 ms. This clear timescale separation supports our conclusion that the final alignment is established predominantly post-scan via diffusion-guided transport and interfacial reorientation. A detailed derivation, assumptions, and supporting measurements are provided in Supplementary Note 8.

R#3-5.2 Discuss whether diffusion-guided reorientation persists for smaller voxel sizes than measured in this work ("449 nm in width and 1495 nm in height") or reduced laser doses.

Response: We thank the reviewer for this question. In principle, diffusion-guided reorientation should still occur at smaller voxel sizes and/or reduced laser doses, because the underlying mechanism—polymerization-induced gradients followed by interfacial diffusion/reorientation—does not require a minimum voxel dimension. In practice, within our material and process window, lowering the dose simultaneously reduces voxel overlap (effective feature continuity) and decreases crosslinking density (network stiffness). These coupled effects weaken both inter-line (hatching,xy-plane) and inter-layer (slicing, z-axis) connectivity and can introduce mechanically fragile interfaces. As a result, although reorientation tendencies may still be present, the 3D architecture becomes prone to instability during stacking and especially during development/drying, where capillary forces can trigger deformation or collapse. Therefore, the practical limit we observe at reduced dose/smaller voxels is governed primarily by geometric continuity and mechanical robustness**,** rather than by the disappearance of diffusion-guided reorientation itself.

Moreover, if the voxel/dose is reduced to the point that polymer conversion becomes too weak to generate a sufficiently strong local concentration (chemical-potential) gradient, the diffusion-driven influx and subsequent interfacial reorientation will also diminish, and the diffusion-guided alignment effect can effectively vanish. Therefore, the practical limit we observe at reduced dose/smaller voxels is governed primarily by geometric continuity and mechanical robustness, and—at more extreme reduction—by an insufficient polymerization-induced gradient, rather than by a fundamental change of mechanism.

We conclude and add description in the revised manuscript (lines 220-223): " *Conversely, reducing dose/voxel size can preserve diffusion-guided reorientation in principle, yet insufficient overlap and stiffness can destabilize 3D stacking, and at extreme reductions polymer conversion may become too weak to sustain the concentration gradients needed for diffusion-driven influx and interfacial reorientation.* "

R#3-5.3 In Fig. 3d, explain the unexpected OPD "bright rim" (~1650 nm → 720 nm → 1650 nm) within the same region; the current interpretation is unclear.

We thank the reviewer for this important comment. We agree that our previous wording did not clearly explain the non-monotonic OPD profile (~1650 → ~720 → ~1650 nm) near the edge in Fig. 3d. We have revised the text to clarify that this feature is attributed to a localized interfacial diffusion-affected shell at the polymer/precursor boundary, rather than a uniform reorientation of the entire in-plane domain. In this narrow interfacial region, diffusion/anchoring competition can induce partial director tilting and/or local changes in effective birefringence/thickness, producing a transient OPD dip (~720 nm), while the interior bulk remains strongly in-plane aligned (~1650 nm). We also note that the post-TPP UV flood cure used to stabilize the sample may induce boundary OPD variations (e.g., the OPD dip at the edge of the LCN micropillar under POM in Fig. 3f). Importantly, this rim is absent in the developed control samples, and therefore any edge-related alignment disturbance is negligible in the final structures.

The description is added in the revised manuscript (lines166 -169): "*We also note that the post-TPP UV flood cure used to stabilize the sample may induce boundary OPD variations (e.g., the OPD dip at the edge of*

the LCN micropillar under POM in Fig. 3f). Importantly, this rim is absent in the developed control samples and therefore the alignment disturbance at the edge is negligible."

6. Long-term actuation stability

R#3-6.1 In Fig. 4b, the actuation amplitude decreases markedly over repeated cycles. Please discuss possible causes (e.g., photothermal fatigue, dye photobleaching, polymer relaxation) and quantify the performance degradation.

Response: We thank the reviewer for pointing this out. The optical actuation in Fig. 4a and 4b is driven by localized laser heating, and the excitation beam inherently follows a Gaussian intensity profile. To reach a contraction level comparable to the thermally saturated strain (~18%), a relatively high peak intensity is required at the beam center; under such conditions we observed localized photothermal damage (and associated irreversible softening/relaxation) near the hotspot, which can manifest as a reduced actuation amplitude upon repeated cycling. Because the deformation is highly sensitive to beam centering and local peak intensity, the laser-driven cycling data are not ideal for quantifying intrinsic fatigue/repeatability of the printed network so we remove the Fig. 4b in the previous manuscript.

To address this limitation and provide a more reliable assessment of stability, we performed repeatability tests using a temperature-controlled heating stage with uniform thermal loading (See the new figure Fig. 4c). We find that the microstrips reach their maximum shrinkage above ~200 °C and exhibit highly repeatable actuation over repeated heating/cooling switching, with consistent peak strain and minimal cycle-to-cycle drift. We have clarified this point in the revised manuscript and now use the thermal-stage results to quantify repeatability and performance stability.

7.Data presentation and figure consistency

R#3-7.1 In several figures (e.g. Fig 5g) the director field is depicted as a vector, pointing in opposite direction with respect to the diffusion. Do the authors mean that mesogenic alignment is polar?

Response: Thank you for pointing this out. To avoid confusion, we have revised the figure by replacing the single-headed arrows with double-headed arrows (↔) to explicitly indicate the non-polar nature of the director field.

R#3-7.2 In Fig. 3e, the bottom-left region unexpectedly brightens after development—explain whether vertical alignment is preserved or altered.

Response: We thank the reviewer for this important comment. The bottom-left region in Fig. 3e corresponds to the vertically aligned supporting/base layer. It is written without an added dwell time, so its orientation is governed primarily by substrate anchoring (vertical alignment) rather than diffusion-driven reorientation. Under our writing conditions, the layer-to-layer time window is short, which is insufficient for full diffusion equilibration before subsequent stacking. As a result, diffusing monomers/mesogens can accumulate near the pillar boundary but are not fully incorporated into the network; these weakly polymerized fractions are preferentially removed during development, causing slight tapering/tilt of the upper part while the bottom remains

fixed to the substrate. This geometry change increases the local OPD and therefore appears brighter in POM. If needed, introducing a short dwell (~3 s) before stacking can improve incorporation near the interface and further suppress this wash-out effect, at the cost of longer writing time.

The description is added in the revised manuscript (lines 169-173): " *In the developed control sample, the vertical region appears grey under POM because the supporting base is written without the inter-layer delay, yielding a lower effective crosslinking density that is more susceptible to development-induced wash-out and slight distortion, which increases the local OPD (dark→grey/bright). It would better preserve the vertical geometry by applying the same inter-layer delay, at the cost of longer writing time.*"

Reviewer #3 (Remarks to the Author):

Summary: Based on an evaluation of the revised manuscript, supporting information, and the rebuttal letter, the authors have improved the technical rigor and linguistic consistency of their work. They have systematically addressed the reviewers' concerns by updating terminology, providing quantitative models, and adding new experimental validations. However, there are still technical improvements to be made before the manuscript before it can be considered for publications in this journal.

R#3-1.• It is recommended that authors submit their manuscripts for thorough review by a colleague who is a native speaker or proficient in academic English. This is due to the fact that some sentences appear to be convoluted and difficult to comprehend.

Response: We thank the reviewer for this suggestion. We have carefully revised the manuscript to improve the clarity and readability of the text, paying particular attention to sentences that may have been convoluted or difficult to comprehend. We believe the revised manuscript now reads more clearly and fluently.

R#3-2. “LC monomer” in Figure 1 and its caption, as well as elsewhere in the text (e.g. Line 341) whenever it is referred to the specific compound should be changed in “RM105”.

Response: We respectfully clarify that the term “LC monomer” has been retained in the revised manuscript. The material employed in this work was purchased from Synthon Chemicals as described in Methods, and the supplier information does not explicitly state that the compound is named RM105. For accuracy and to avoid unsupported identification of the material, we therefore use the more general term “LC monomer” throughout the manuscript.

R#3-3. Line 46. Please check “volumetric4D volumetric”.

Response: We thank the reviewer for catching this. We have revised the

redundant phrase "volumetric4D volumetric" to "volumetric 4D microprinting" at Line 47 in the revised manuscript, which now reads: "leaving volumetric 4D microprinting with programmable, arbitrary 3D director fields an unresolved challenge."

R#3-4. Line 92. “scanning along the x-axis 0.2 micron”. In Fig. 2 laser is scanned along the y-axis. 0.2 micron should be removed.

Response: We thank the reviewer for this comment. We have removed the original sentence at Lines 84–85 and replaced it with: "For example, scanning along the y-axis with a lateral step (hatch) of 0.2 μm along the x-axis forms a continuous, structurally coherent LCN layer (Fig. 2)."

R#3-5. Lines 126-127. In reviewer’s opinion, “y-direction” and “y” should be changed into “x-direction” and “x” as the polymerized ribbons are parallel to the x-axis.

Response: We thank the reviewer for this comment. We would like to respectfully clarify that the LC alignment direction is determined by the scanning direction. When voxel scanning is performed along the x-direction, mesogen diffusion occurs toward the sidewalls of the polymerized lines, which are perpendicular to the scanning direction, resulting in LC alignment along the y-direction. Conversely, scanning along the y-direction yields LC alignment along the x-direction. This relationship is the key design principle we wish to convey. We have revised Lines 118-120 accordingly to make this distinction clearer: "...i.e., performing voxel scanning along the x-direction promotes mesogen diffusion toward the sidewalls of the polymerized lines, resulting in LC alignment along the y-direction."

R#3-6. Lines 169-170. In the reviewer’s understanding the supporting base is a single layer. How the "inter-layer" delay should apply in this case?

Response: We thank the reviewer for this comment. We clarify that the supporting base is not a single layer but rather the vertically aligned region of the dual-aligned pillar, comprising multiple stacked layers written without an inter-layer delay, since the substrate-imposed homeotropic anchoring already dictates the desired vertical orientation without requiring diffusion-guided

reorientation. However, since the horizontally aligned region is written with an inter-layer delay, a difference in crosslinking density arises between the two regions. This mismatch leads to slight structural deformation after development, increasing the local OPD and causing the vertical region to appear grey rather than dark under POM (dark→grey). Importantly, the LC alignment itself is not affected by this crosslinking density difference — the grey appearance is purely a post-development geometric artifact. Therefore, for applications where the structure does not require development, the LC alignment is fully preserved and unaffected. For applications requiring precise geometry preservation after development, we recommend applying the same inter-layer delay to the vertical region to maintain crosslinking consistency, at the cost of slightly longer writing time. We have revised Lines 151–155 accordingly to make this distinction explicit: " In the developed sample, the vertical region of the dual-aligned pillar appears grey under POM because it is written without the inter-layer delay, yielding a lower effective crosslinking density that is more susceptible to development-induced wash-out and slight distortion, which increases the local OPD, causing the region to appear grey or bright rather than dark. Applying the same inter-layer delay to the vertical region is recommended to preserve geometric accuracy, at the cost of slightly longer writing time."

R#3-7. Line 180. “line” should be “layer”, as 3s is the inter-layer delay.

Response: We have revised “line” to “layer” in the revised manuscript at Line180, which now reads "... before each layer is written." at Lines 161-162 in the revised manuscript.

R#3-8. Line 190. Please rephrase “….during the voxel scanning during the change…”

Response: We have removed the original sentence at Lines 474 and replaced it with: “Schematic of diffusion-induced alignment during voxel scanning as the stacking direction changes.” in the revised manuscript.

R#3-9. Line 192. The sentence “d” is not complete: “micropillar before ……” ?

Response: We have removed the original sentence at Line 476 and

replaced it with: “POM image of the dual-aligned micropillar before development”.

R#3-10. Line 202 and beyond. “strain of about 17.4%, with 17.1% already achieved”. The authors here refer to the change in length of a 55 micron microstrip, for which a 0.1% variation corresponds to 55 nm, well beyond the optical resolution in Fig. 4a. The authors should consider the true measurement uncertainty.

Response: We agree with the reviewer that the originally reported values were overly precise given the limited image resolution of the POM measurements. Therefore, the relevant values have been re-examined and corrected accordingly, with the change in length reported to one decimal place and the corresponding contraction strain expressed as an integer percentage. The corresponding revisions are summarized below.

1. Lines 174–176 have been revised to “Upon heating to 230 °C, the microstrip exhibited rapid and reversible contraction along the mesogen alignment direction, with the contraction strain remaining at ~17% above 200 °C (Fig. 4b).”

2. Line 182 has been revised to: “…..approximately 17% over 24 consecutive …..” in the revised manuscript.

3. Line 190 has been revised to: “achieved ~17% actuation strain falls…..” in the revised manuscript.

4. Line 483 has been revised to: “……,reaching a maximum contraction strain of ~17%.” in the revised manuscript.

R#3-11. Inappropriate use of significant digits occurs also in the voxel dimensions. The authors state that the resulting voxel dimensions were measured to be “approximately 449 nm in width and 1.495 μm in height”. Reporting a voxel dimensions to the nearest nanometer is unrealistic for experimental DLW characterization. Typical laser-written voxels are subject to fluctuations in laser power, local resin heterogeneity, and polymerization shrinkage, which generally limit repeatable precision to the 10–50 nm range at

best.

Response: We agree with the reviewer that the originally reported voxel dimensions were overly precise for experimental DLW characterization. To reflect a more realistic precision range, the values have been rounded throughout the revised manuscript and Supplementary Information: 449 nm has been revised to 0.45 μm, and 1.495 μm has been revised to 1.5 μm.

R#3-12. Line 209. “differencing” should be “different”

Response: We have revised to “…events (12 heating/cooling cycles) for different…” at Line 182-183 in the revised manuscript.

R#3-13. Line 230. “R” should probably be “RT”.

Response: We have revised to “RT” at Line 485 in the revised manuscript.

R#3-14. Line 235. “blue region” should become “pink region”

Response: We thank the reviewer for this correction. We agree that the original "blue region" required revision. Furthermore, as pink and green can be difficult to distinguish for readers with color-vision deficiencies, we have ensured that all colors are clearly labeled and defined in the figure legends. Accordingly, the figure colors and corresponding text have been updated, with the sentence now reading: "Schematic of DLW at the interface: the green region represents the first-written polymerized domain, while LC mesogens from the surrounding unpolymerized region diffuse into the green domain and are subsequently polymerized by line-by-line laser scanning (pink color)." at Lines 489-491 in the revised manuscript.
